# Caregiving experiences and relationships among migrant care workers, older care recipients, and employer families: a scoping review

**DOI:** 10.1093/geront/gnaf300

**Published:** 2025-12-15

**Authors:** Igone Etxeberria, Fátima María García-Pena, Maite Azabal, Karl Pillemer

**Affiliations:** Department of Clinical and Health Psychology and Research Methods, Faculty of Psychology, University of the Basque Country (UPV/EHU), Donostia, Spain; Department of Clinical and Health Psychology and Research Methods, Faculty of Psychology, University of the Basque Country (UPV/EHU), Donostia, Spain; Department of Social Psychology, University of the Basque Country (UPV/EHU), Donostia, Spain; College of Human Ecology, Cornell University, Ithaca, New York, United States; Division of Geriatrics and Palliative Medicine, Weill Cornell Medicine, New York, United States

**Keywords:** Caregiving dynamics, Home care, Triad, Foreign care workers

## Abstract

**Background and Objectives:**

This scoping review explored caregiving experiences and relationships among migrant home care workers, older adult care recipients and employer families, with the aim of analyzing the nature and extent of these relationships and understanding the factors that influence caregiving dynamics.

**Research Design and Methods:**

The review was conducted in accordance with Arksey and O’Malley’s scoping review framework. The researchers searched WoS Core Collection, Scopus, Pubmed, EBSCOhost: Psychology and Behavioral Sciences, EBSCOhost: PsycArticles, EBSCOhost: PsycInfo databases, and Google Scholar. Inclusion criteria were (a) articles reporting a research study; (b) articles focusing on triadic or dyadic caregiving relationships and experiences involving the migrant home care worker and/or the older care recipient and/or the employer family; (c) articles focusing on transnational migrant care workers; (d) articles published since 2013; and (e) articles written in English or Spanish. The researchers screened a total of 637 records, and 38 articles met the inclusion criteria.

**Results:**

These studies represented a number of countries and used various quantitative, qualitative and mixed-method study designs. The findings revealed that caregiving dynamics among the three parties are strongly influenced by cultural, structural, and interpersonal factors. Positive relationships improve the well-being of all parties involved; however, challenges such as abuse, exploitation, and poor working conditions persist, especially for live-in care workers.

**Discussion and Implications:**

Strengthening triadic caregiving relationships through cultural training, improved legal and institutional protections and psychosocial and language support are critical for enhancing quality of care and the well-being of all those involved in the caregiving process.

Older adults often wish to be cared for in their own homes (Ayalon, 2009a). Traditionally, home care has been provided by family members, and primarily women. However, increasing life expectancy, decreasing birth rates, increasing geographical distance between family members, and the greater prevalence of women in the workforce have resulted in families being less able to provide informal care (Harrington, 2000). Moreover, the feminization of international migration over recent decades, combined with a decline in state-provided care services, has contributed to a global shift in the responsibility for caring for older adults in the community from family members to live-in migrant care workers (Bauer & Österle, 2013). This phenomenon is particularly prevalent in Europe, North America, the Middle East, and Asia (Cohen-Mansfield et al., 2013; van Hooren, 2012).

Foreign or migrant domestic workers are mostly young women from low-and middle-income countries (LMICs), commonly employed as live-in caregivers for older individuals or those requiring home care (Basnyat & Chang, 2017). These migrant domestic workers usually perform both caregiving and domestic tasks, including cooking, cleaning, and caring (Anderson, 2000). It is estimated that there are a total of 11.5 million migrant domestic workers worldwide, although figures vary widely across countries, with the Arab States, North America, and Southern and Western Europe collectively accounting for approximately 52% of this population (Galloti, 2015).

Migrant care workers’ experiences vary significantly across countries, influenced by host nations’ welfare state and immigration policies (van Hooren, 2012). In Northern Europe, Canada, and the United States, migrant care workers often work in the professional care sector, whereas in Southern Europe, the Middle East, and parts of Asia (e.g., Singapore), they are primarily employed privately as live-in caregivers (Munkejord et al., 2021b). Some countries, such as Canada and Hong Kong, regulate the recruitment and working conditions of migrant domestic workers through formal labor programs (Hondagneu-Sotelo, 2001). Taiwan mandates agency employment and pre-placement training for live-in caregivers, although these workers face certain restrictions (such as limited stays and the inability to change employers unless the care recipient dies) and lack protection under labor laws (Liang, 2014; Munkejord et al., 2021a). Similarly, in Germany, live-in migrant care workers operate outside the formal long-term care system under a market-driven model, in which agencies mediate arrangements between families and migrant care workers. A 2021 ruling by the German Federal Labour Court affirmed that live-in caregivers are entitled to a minimum wage for all working hours, including on-call time, day and night (Grenz & von Kutzleben, 2024).

Israel’s 1988 Long-Term Care Community Law (LTCCL) supports older adults at home, with 70% using LTCCL-funded home care services (National Insurance Institute of Israel, 2011, cited in Ayalon & Roziner, 2016). These services include live-out care by local workers for limited hours and round-the-clock care by temporary migrant care workers, primarily from East Asia and Eastern Europe, whose stay is restricted to a maximum of five years or until their employer’s death (Ayalon et al., 2013). In contrast, the United States lacks formal systems for recruiting foreign domestic workers (Hondagneu-Sotelo, 2001).

In Southern Europe, especially Greece, Italy, and Spain, female migrants are increasingly replacing native women in informal care roles, creating a new labor division involving families, the market, and the state. This shift reflects a transition from family care to a “migrant in the family” model, allowing older adults to remain at home, albeit in often irregular arrangements (Bettio et al., 2006). Díaz-Gorfinkiel and Martínez-Buján (2018) call this a “modified family-social structure” because, although care is outsourced, older individuals are still cared for at home and care is provided by women.

Previous research has shown that introducing a live-in migrant care worker into the family changes its dynamics. Employer families maintain some of their caregiving tasks, mainly acting as care managers and mediators between the older care recipient and the migrant care worker. For their part, migrant care workers take on both emotional and nursing duties (Ayalon, 2009a). The home becomes a private workplace and the blurred boundaries between home and work shape the relationship established among live-in migrant care workers, employer families, and care recipients.

Several studies have documented the importance of the close ties that are formed between older adults, their family members, and the migrant care workers (Ayalon, 2009a; Iecovich, 2016; Parreñas, 2014). Salami and Meherali (2018) found reciprocity and respect to be important ingredients for healthy relations between live-in migrant care workers and employer families, because without these qualities, relationships are more likely to become exploitative due to the unequal power dynamics inherent to the employer–employee relationship. Moreover, families often have negative stereotypical beliefs about live-in migrant care workers and may, on occasions, abuse and exploit them (e.g., Cheung et al., 2019), although abuse and neglect of care recipients by live-in caregivers have also been documented (e.g., Ayalon, 2009a, b, 2011a).

Despite the importance of these relationships and their influence on care satisfaction, the dynamic interactions between employer families, older care recipients and migrant care workers remain under-investigated. Salami et al. (2017) conducted a scoping review focusing on the perspectives of employer families of live-in migrant care workers, and other studies have focused on the perspective of older adults (Shinan-Altman & Ayalon, 2019), live-in migrant care workers (Septi-Mauludina et al., 2023), or both (Yeoh et al., 2023). Nevertheless, in the context of the growing global trend toward older adults being cared for by live-in migrant care workers, with families assuming employer roles, further research is required to explore the dynamics of caregiving experiences and relationships among all three parties. This knowledge could ultimately help improve the quality of care provided within this arrangement.

This study aims to analyze the extent and nature of the caregiving experiences and relationships between migrant care workers and/or older adult care recipients, and/or employer families. Specifically, the aim is to answer the following question: What are the major findings in the international literature regarding the caregiving experiences of, and the relationships between migrant care workers and/or care recipients, and/or employer families?

## Method

### Study design

Given the breadth of the research question, a scoping review approach was adopted to map the relevant literature in this field. A scoping review provides greater conceptual clarity about a specific topic, rather than assessing the quality of existing studies (Arksey & O’Malley, 2005). The review followed Arksey and O’Malley’s (2005) five-stage framework for scoping reviews. The researchers identified the research question (stage 1), then developed a search strategy for the identification of relevant studies (stage 2), conducted the study selection (stage 3), then charted the key findings from these studies using thematic analysis (stage 4), and finally collated, summarized, and reported the results (stage 5). In addition to Arksey & O’Malley’s (2005) framework, the most recent methodological updates were also considered (Levac et al., 2010; Peters et al., 2020, 2021; Pollock et al., 2023) and adhered to the Preferred Reporting Items for Systematic Reviews and Meta-Analyses extension for Scoping Reviews statement guidelines (Tricco et al., 2018). However, the researchers also integrated the updated guidelines from the recent PRISMA 2020 Statement (Page et al., 2021) for reporting systematic reviews, which provides valuable instructions pertaining to several aspects that both types of review have in common (e.g., the new flow diagram design). The scoping review was registered in Open Science Framework (https://osf.io/f78j2/overview).

### Eligibility criteria

The following inclusion criteria were applied: (a) articles reporting a research study (the researchers excluded scoping reviews, systematic reviews, doctoral theses, case studies, etc); (b) articles focusing on triadic or dyadic caregiving relationships and experiences involving migrant care workers and/or older care recipients and/or employer families (in cases in which only one party was involved in the study, the article was only included if the study addressed relationships with, or caregiving experiences involving, other parties); (c) articles featuring transnational migrant workers employed as live-in and live-out caregivers; (d) articles published between January 2013 and September 2025; and (e) articles written in English or Spanish. The exclusion criteria were: (a) care was not provided at home; (b) the migrant care workers in question did not care for older adults (cases in which care was provided to individuals under 60 years of age and children); (c) migrant care workers only performed household chores; and (d) full text was not available.

### Information sources and search strategies

The databases searched were WoS Core Collection, Scopus, Pubmed, EBSCOhost: Psychology and Behavioral Sciences, EBSCOhost: PsycArticles, and EBSCOhost: PsycInfo. The search was conducted in September 2025 by the first two authors. The asterisk (*) wildcard symbol was used to encompass all keyword variations stemming from the same root, thus expanding the search. The keywords and the databases were selected in collaboration with a research support librarian to ensure that the search included the relevant databases. In addition to electronic database searches, and with the aim of exploring gray literature as well as the identification of potentially relevant studies that might not be captured by the cited databases searches, a Google Scholar search was also conducted (Bramer et al., 2017; Haddaway et al., 2015). Finally, reference lists were also scanned. The complete search strategy is outlined in Table 1 and Supplementary Material 1 (see online supplementary material).

**Table 1.** Search terms used.

	Search terms
**1**	“Migrant Home Care*”
	“Foreign Home Care*”
	“Migrant Homecare*”
	“Foreign Homecare*”
	“Migrant Home Care Worker*”
	“Foreign Home Care Worker*”
	“Migrant Home Worker*”
	“Foreign Home Worker*”
	“Migrant Homeworker*”
	“Foreign Homeworker*”
	“Migrant Domestic Care*”
	“Foreign Domestic Care*”
	“Migrant Domestic Care Worker*”
	“Foreign Domestic Care Worker*”
	“Migrant Domestic Worker*”
	“Foreign Domestic Worker*”
	“In-Home Migrant Care*”
	“In-Home Foreign Care*”
	“In-Home Migrant Care Worker*”
	“In-Home Foreign Care Worker*”
	“In-Home Migrant Worker*”
	“In-Home Foreign Worker*”
	“In-Home Paid Migrant Care*”
	“In-Home Foreign Paid Care*”
	“In-Home Paid Migrant Care Worker*”
	“In-Home Paid Foreign Care Worker*”
**2**	“Family Care*”
	“Employer Famil*”
	“Informal Care*”
**3**	“Elderly”
	“Older Adult*”
	“Care Recipient”
	“Dementia*”
	“Alzheimer*”
	“Dependen*”
**4**	#1 AND #2
**5**	#1 AND #3
**6**	#4 OR #5

*Note.* In the Google Scholar database, underlined terms were removed due to lack of results.

### Study selection

The records obtained from the databases and Google Scholar were imported into an Excel spreadsheet and manually removed the duplicates by two members of the team (I.E. and F.M.G.P.). The resulting records were analyzed in a two-phase screening process using previously set eligibility criteria to determine their inclusion in the review. The first screening of the title and abstract was conducted in pairs by the same authors. Each reviewer worked independently at first, and then the pair met to discuss their evaluation. Any disagreement or uncertainty was resolved by consensus between the reviewers. If needed, a third reviewer was involved (M.A.) to avoid principal researcher bias. In this first step, where necessary, the full texts of some articles were also read, mostly in cases in which the relevant information was not clearly stated in the title and abstract. In the second step, the full texts of the articles were scanned, and the reference lists reviewed, with any articles deemed eligible being included. The procedure followed was the same as in the first step.

### Data extraction

Basic descriptive data were extracted from the studies by the authors to a table containing the following information: Author/year, country, purpose/aim, methods, participants, major findings, and theme(s). The first three authors reviewed and independently verified the data extraction process, and several meetings were held to discuss the results. The last author reviewed all the data extraction process.

### Data analysis and presentation

The results of this scoping review are presented in Table 2, which outlines the number of included studies, the variety of study designs employed, the countries in which studies were conducted, and major findings. Thematic analysis is a methodology used to identify, analyze, and report recurrent patterns of meaning (themes) within a dataset (Braun & Clarke, 2006). It is a set of methods extensively used for developing and analyzing patterns of meaning across qualitative data (Braun & Clarke, 2022). The data analysis process involves becoming familiar with the dataset, generating initial codes, searching and developing preliminary themes, reviewing and refining identified themes, defining and labeling themes, and writing the final report (Braun & Clarke, 2006).

**Table 2.** Characteristics of the included studies.

Authors/year	Country	Purpose/aim	Methods	Participants	Major findings	Theme(s)
**Ayalon (2014** **)**	Israel	To analyze the impact of familiarity and satisfaction when comparing migrant care workers to Israeli caregivers as predictors of attitudes toward social rights for foreigners.	Quantitative Self-reports Five items on a seven-point scale assessed attitudes toward social rights for foreigners, covering health, education, welfare, living arrangements, and minimum wage. The HCSM included 13 questions on satisfaction with home care.	388 older adults and 686 employer families. 666 with migrant care workers and the remaining sample with Israeli home care workers.	Satisfaction with services and attitudes toward rights for foreigners are more strongly associated with each other among those hiring migrant care workers than among those relying on Israeli home caregivers. Positive relationships with migrant care workers also foster favorable attitudes. In addition, higher socioeconomic status within families, which facilitates the hiring of foreign care workers, may correlate with more positive attitudes toward foreigners.	Relational dynamics: quality of the relationships (migrant care workers-employer families)
**Ayalon (2016** **)**	Israel	To examine the characteristics of caregivers and care recipients as potential predictors of elder neglect, considering the perspectives of care recipients, employer families, and migrant care workers.	Quantitative Self-reports. Neglect was assessed on a 6-item scale, addressing both unmet needs for services and unmet needs for assistance in ADL or IADL tasks.	338 older adults, 523 migrant care workers and 689 employer families.	31.5% of older adults, 18% of migrant care workers, and 32.3% of family members reported some form of elder neglect. Live-in caregiving is linked to lower family caregiver burnout and less neglect compared to live-out care. While informal caregiver burden does not directly impact neglect, migrant care worker burden is directly related. Greater collaboration with family employers helps reduce migrant care workers’ burden.	Job satisfaction, abuse and neglect Well-being: migrant care workers and employer families
**Ayalon et al. (2025** **)**	Israel	To examine the perspectives of family members and migrant home care workers concerning dementia care.	Qualitative study Interviews about migrant care workers and family caregivers’ perception of dementia and its accompanying behavioral problems, strategies used, and how dementia and its accompanying challenges are addressed within the caregiving context.	21 migrant care workers and 17 family caregivers who care for people with dementia.	The main themes identified were: (a) challenges associated with caring for a people with dementia; (b) management of the care recipient’s behavioral problems; and (c) relationship between family members and the migrant care worker.	Relational dynamics: satisfaction and quality of relationship (migrant care workers–employer families, migrant care workers–care recipients)
**Ayalon and Roziner (2016** **)**	Israel	To explore relationship satisfaction from the perspective of all three members of the home caregiving triad: (a) exploring which member of the triad has the most impact on the range in reported satisfaction; (b) describing the level of reciprocity in individual satisfaction ratings; and (c) exploring the level of satisfaction reciprocity among the three parties.	Quantitative Cross-sectional design. Interviews with one, two, or three members of the caregiving triad. Variables: (a) Satisfaction with relationships: each member reported how satisfied they were with all the other members of the unit. (b) The care recipient’s functional status, capacity to execute six ADL.	223 complete caregiving triads made up of an older adult, a family member, and a migrant care worker.	The variability linked to the migrant care worker as a partner was significantly higher than that linked to the older adult or family member. These results highlight the key role of the home care worker in overall satisfaction within the caregiving triad.	Relational dynamics: satisfaction with relationships (triad)
**Basnyat and Chang (2017** **)**	Singapore	To investigate family caregivers’ relationship with migrant live-in care workers and its impact on coping with caregiver burden.	Qualitative study In-depth interviews with 20 employer families.	18 female and 2 male caregivers, providing care for an average of 6 years.	Caregivers view hired support as a helpful strategy to reduce physical strain, relieve stress, and create personal time for leisure and work. They also offer support to migrant care workers through training, empathy, and advice.	Well-being: employer families
**Cohen-Mansfield and Golander (2021** **)**	Israel	To identify factors predicting the burden/burnout and job satisfaction of live-in migrant care workers. To develop models linking care workers’ burden/burnout, job satisfaction, and predictors such as work-related and employment-related factors.	Quantitative Structured interviews with live-in migrant care workers. Outcomes: (a) care worker’s job satisfaction and (b) level of burden: 5 items from the ZBI and Burnout Scale. Independent variables: (a) older person’s degree of disability; (b) care worker’s relationship with the older person; and (c) employment conditions and their satisfaction with them.	116 migrant care workers providing care for older individuals in Israel. Mostly females (91.9%), with an average age of 38.8 years.	Work-related factors (care recipient’s functional status and the worker’s relationship with them) directly impacted burnout, whereas employment-related factors (conditions and satisfaction) had an indirect effect. Migrant care workers’ job satisfaction was influenced by both work dynamics (relationship with the care recipient) and their response to employment conditions.	Job satisfaction, abuse and neglect Well-being: migrant care workers (burden and burnout)
**Cohen-Mansfield and Golander (2022)**	Israel	To explore the relationship between caregiver-centered factors associated with employment conditions and the quality-of-care provided by migrant live-in caregivers to older care-recipients	Quantitative Cross-sectional study Structured interviews with migrant live-in caregivers, older care recipients, and their relatives.	115 migrant live-in caregivers, 72 older care recipients, and 117 relatives of care recipients. In total, 91 relative–caregiver dyads; 53 care–recipient–caregiver dyads, and 36 triads.	The quality of the relationship between the older care recipient or relatives of the care recipient) and live-in migrant care worker directly predicted quality of care. Care quality was most strongly and consistently predicted by the quality of the relationship between migrant care worker and the older care recipient, as well as the migrant care worker and the relative. Factors associated with employment conditions had also a significant indirect effect on quality of care. Quality of the relationship also improved significantly when care recipients were female and needing more assistance with daily activities.	Relational dynamics: quality of relationship (migrant care workers–employer families, migrant care workers–older adults)
**Cohen-Mansfield and Golander (2023** **)**	Israel	To gain better understanding of the formal and informal nature of the relationship between family carers and migrant care worker.	Mixed-methods Quantitative They were asked to rate the quality of their relationship (family–migrant care worker and Family/migrant care worker care recipient) on a 6-point scale. Qualitative Structured interviews following grounded theory framework	116 migrant care workers and 117 family caregivers, including 92 dyads.	Quantitative findings showed a significant correlation between migrant care workers’ and family caregivers’ ratings of relationship quality, with families rating it more positively. Migrant care workers’ ratings of their relationships with family caregivers were significantly correlated with the quality of their relationships with the care recipients. In the qualitative analysis, four main themes emerged: (a) initial and ongoing interdependence; (b) communication; (c) perceived relationship between family caregivers and migrant care worker; and (d) triadic relationship: family caregiver-migrant care worker–care recipient.	Relational dynamics: quality of the relationships (migrant care workers–employer families) Relational dynamics: satisfaction with relationships (triad)
**Goldzweig et al. (2015)**	Israel	To explore and make recommendations to oncologists regarding the distress experienced by migrant care workers to older adult cancer patients.	Quantitative study Individual interviews with migrant care workers addressing feelings toward the care recipient family; the level of experienced distress measured by the Distress Thermometer; the perception of the distressing impact of caregiving; and basic sociodemographic questions.	108 Filipino migrant care worker providing care at home to persons aged 65+ (55 caring for cancer patients, and 53 caring for older adults with other chronic diseases).	Participants reported extreme levels of distress. Migrant care workers of older adults cancer patients reported significantly higher levels of distress and negative caregiving impact in comparison to caregivers of patients with other chronic illnesses. They felt less attached to the care recipient’s family (less loved by and less taken care of).	Relational dynamics: satisfaction and quality of relationship (migrant care workers-employer families) Well-being (migrant care workers)
**Green and Ayalon (2015** **)**	Israel	To explore older adults’, family members’ and live-in migrant care workers’ level of knowledge about the rights of migrant live-in care workers.	Qualitative study using questionnaires. Interviews assessed sociodemographic characteristics (age, gender, financial situation) and familiarity with migrant care workers’ rights using a 10-item questionnaire on fundamental worker rights, rated true or false.	338 live-in migrant care workers, 224 older adults over the age of 70, and 442 family members. 84% of migrant care workers were women, and 44.4% were from the Philippines.	Knowledge of workers’ rights among live-in migrant care workers was moderate. On average, older adults knew 4.22 out of 10 rights, while family members and migrant care workers knew 7.77 and 7.34, respectively. Comprehensive knowledge of all rights was limited, with only 4% of older adults, 18% of family members, and 10% of migrant care workers being fully aware.	Job satisfaction, abuse, and neglect
**Green and Ayalon (2017** **)**	Israel	To identify the characteristics of migrant care workers, care recipients, and work conditions linked to work-related abuse of migrant care workers in Israel.	Quantitative Questionnaire Sociodemographic; Work environment; Care recipient’s cognitive state; Exposure to abuse and exploitation.	187 Filipino live-in migrant care workers. 86.8% women.	55% of migrant care workers reported work-related abuse, including sexual, physical, and/or emotional mistreatment and exploitation, such as performing tasks beyond their job requirements. One-third of these incidents occurred with their current employer, with emotional and physical abuse being the most common. Financial dependence often prevents migrant care workers from leaving abusive workplaces. Those caring for cognitively-impaired older adults experienced higher abuse rates. Longer employment periods were linked to less abuse, whereas larger households correlated with more abuse. Male care workers faced a higher risk of abuse than females.	Job satisfaction, abuse, and neglect
**Green and Ayalon (2018** **)**	Israel	To assess working conditions and the prevalence of abuse and exploitation among live-in migrant care workers and local live-out care workers.	Quantitative 338 live-in migrant care workers and 185 local live-out home care workers participated in a face-to-face survey. Measures: Socio demographics; Violation of workers’ rights; Exposure to work-related abuse; Living conditions.	Live-in migrant care workers: 84% women, mean age: 38 years; 58% married; 62% were high-school graduates. Local live-out home care workers: 92.2% women; mean age: 53 years; 56.2% married; 70% high-school graduates.	58% of live-in migrant care workers had no vacation days beyond their weekly day off, and 30% did not consistently receive a day off. 79% lacked paid sick days, and 15% did not have a contract. 12.7% reported emotional abuse, while 1% experienced physical or sexual abuse. Additionally, 12.3% did not have their own room, and 3.7% lacked their own bed.	Job satisfaction, abuse, and neglect
**Grenz and von Kutzleben (2024** **)**	Germany	To examine how family caregivers of people with dementia conceptualize good live-in migrant care and conflicts that may arise in live-in care arrangements when expectations are not met	Qualitative-explorative approach Online focus groups carried out as semi-structured interviews with 15 employer families. Five focus groups were conducted with two or three participants, plus one individual interview	15 German employer families of care-dependent people with dementia.	Seven main subcategories emerged: (a) assurance of continuity of care in a familiar and safe environment; (b) the ability of the live-in carer to learn about the needs and preferences of the care recipient; (c) the promotion and preservation of resources and social participation of the migrant care workers; (d) allowing spaces for mutual closeness and for sharing emotions; (e) lack of fit or mismatching; (f) intransparency and unreliability in the work of placement agencies; and (g) issues with instability and permanent crisis of the German care work model.	Relational dynamics: satisfaction and quality of relationship (migrant care workers-employer families)
**Heng et al. (2019** **)**	Singapore	To investigate the caregiving experiences and coping strategies of migrant care workers who care for older adults in Singapore.	Qualitative study 11 in-depth interviews with migrant care workers.	Females. Mean age: 35.40 years. Nationality: 1 Burmese, 4 Filipino and 6 Indonesian. 3 live alone with their care-recipient.	Four key themes emerged: (a) balancing caregiving with other duties; (b) overcoming obstacles like language barriers and behavioral challenges; (c) using coping strategies such as time management and self-distraction; and (d) Seeking support from employers, families, and healthcare professionals.	Relational dynamics: satisfaction with the relationships (migrant care workers-older adults) Job satisfaction, abuse, and neglect Well-being: migrant care workers (coping strategies)
**Ho et al. (2018** **)**	Hong Kong	To examine the lived experiences of migrant care workers working with older people living in the community of Hong Kong.	Qualitative study Interviews with 11 migrant care workers.	11 Indonesian and Filipino migrant care workers	The main themes identified were: (a) migrant care workers as inescapable functioning commodity; (b) reciprocity of companionship; and (c) the waxing and waning between the companionship.	Relational dynamics: types of relationship (migrant care workers–employer families, migrant care workers–older adults)
**Ho et al. (2019** **)**	Hong Kong	To explore live-in migrant care workers’ emotional labor, focusing on: (a) methods for regulating emotions with older adults; and (b) the role of morality in emotional labor.	Qualitative study 11 live-in migrant care workers were interviewed in unstructured interviews.	7 Filipino and 4 Indonesian.	Emotional labor, involving the regulation of emotions, poses a moral challenge for live-in migrant care workers amid socio-cultural pressures. Despite this, authentic emotional connections drive them to advocate for older adults’ welfare. By balancing genuine and strategic emotional expressions, migrant care workers use emotional labor to navigate moral interactions.	Well-being: migrant care workers (emotional management)
**Ho et al. (2022** **)**	Hong Kong	To determine the prevalence of loneliness among community-dwelling older adults. To analyze the link between co-living with migrant care workers and loneliness in this population.	Quantitative Interviews guided by questionnaires. Outcomes: Chinese version of the 6-item DJGLS; Living or not living with a migrant care worker. Cofunders: Sociodemographic; 10-item LSNS, Chinese version; Number of chronic illnesses; AMT, Hong Kong version; Lawton’s IADL scale Hong Kong Chinese version.	380 older adults aged 60 years or over. Mean age: 70.8 years. 51.3% female.	35.3% of older adults reported moderate-to-severe loneliness. Co-living with migrant care workers was linked to reduced overall and emotional loneliness, although not social loneliness.	Well-being: older adults
**Ho et al. (2023** **)**	Hong Kong and United Kingdom	To examine the quality of older adults’ relationships with migrant care workers. To analyze the link between perceived loneliness and the quality of these relationships.	Quantitative Cross-sectional study. Variables studied: (a) loneliness: 6-item DJGLS; (b) the quality of the relationships: 15-item Mutuality Scale.	178 community-dwelling older adults [mean age: 83.44 (SD 7.05 years); 155 (87.1%) women and 23 men (22.9%)], supported by migrant care workers.	Participants reported high levels of social and overall loneliness, linked to poor relationship quality with migrant care workers, although no correlation was found with emotional loneliness.	Relational dynamics: satisfaction with the relationships (migrant care workers-older adults)
**Hoens and Smetcoren (2023** **)**	Belgium	To analyze the motivations of older adults and their families to hire a live-in migrant care worker, and to examine their perception about the presence and care of a live-in migrant care worker.	Qualitative study Individual interviews with older adults or their informal caregivers who hired live-in migrant care worker and focus group or individual interview with professionals whose jobs involved contact with older people who relied on live-in migrant care workers.	8 older adults or their family members, 6 health professionals participated in a focus group, and 5 health professionals and staff members participated in individual interviews.	Two main themes emerged: (a) the motivations of the older adults and their informal caregivers to hire a migrant care worker; and (b) the experiences arising from the presence and care of a live-in migrant care worker.	Relational dynamics: satisfaction and quality of the relationship (migrant care workers–employer families, migrant care workers–older adults) Job satisfaction, abuse, and neglect
**Hung et al. (2024** **)**	Hong Kong	To explain how the quality of dyadic relationships with migrant care workers is associated with older adult’s loneliness and further explore older adults’ perceived experiences of cared by migrant care worker.	Mixed-methods A total of 288 older adults (≥60 years old) living with migrant care workers completed a survey collecting the following information: sociodemographics; IADL; Loneliness; Mutuality; Social network. 19 were in –depth interviewed	288 older adults over 60 years old living with and cared for by migrant care workers for daily living. 19 of them were in-depth interviewed	44% of older adults felt lonely and had social isolation with reduced social engagement with their family and friends (50.7%). They reported poor mutuality with their migrant care workers, and social loneliness negatively and significantly correlated with perceived dyadic relationship quality and social network. Two themes emerged: (a) established relational interaction with migrant care workers; and (b) enjoyed functional assistance and support.	Relational dynamics: satisfaction and types of relationships (migrant care workers–older adults) Well-being: older adults
**Iecovich (2016** **)**	Israel	To assess ambivalent relationships between older care recipients and their family caregivers and migrant care workers. To explore how ambivalence influences loneliness among care recipients.	Quantitative 279 triads (care recipients, their primary family caregivers and migrant care workers) were interviewed face-to-face. Loneliness: JGLS; Ambivalence: DRS; Self-related health; Perceived economic status; Frequency of meeting with children; Length of care and care hours; Sociodemographic.	Care recipients: 69.5% women; Mean age: 83.87 years. Primary family caregivers: 58.2% women; Mean age: 57.85 years. Migrant care workers: 85.5% women; Mean age: 35.98 years.	Ambivalence in relationships with family caregivers and migrant care workers negatively correlated with loneliness. Positive correlations were found for dyadic ambivalence between care recipients and both caregivers. Better health and economic status, along with reduced ambivalence with family caregivers (but not migrant care workers), lowered loneliness levels.	Relational dynamics: Satisfaction with the relationships (triad) Well-being: older adults
**Kaur-Gill and Dutta (2021** **)**	Singapore	To explore how migrant care workers perceive and experience mental health within their role as foreign domestic workers in Singapore.	Qualitative study 20 in-depth, unstructured interviews with domestic workers focused on their perceptions of mental health.	20 migrant care workers from diverse regions of Southeast Asia who collaborated with a local NGO as part of a six-year ethnographic study.	Participants described living in tightly controlled environments, facing emotional stress, abuse, neglect, and limited access to information, all contributing to their marginalization.	Job satisfaction, abuse, and neglect Well-being: migrant care workers (coping strategies and mental health)
**Kriegsmann-Rabe et al. (2023** **)**	Germany	To analyze the stressors and factors that promote the well-being and psychological resilience of live-in migrant care workers.	Qualitative study 16 in-depth interviews with migrant care workers.	Polish live-in care workers: 15 female and 1 male. Ages 36-68; Mean of 12.6 years of work experience as a live-in caregiver in Germany. Worked without an official contract (n = 1), for a brokerage agency (*n* = 1), self-employed (*n* = 4) and family-employed (*n* = 9).	Stressors: (a) interactions with care recipients (illness, lack of empathy/trust); (b) relationships with family members (rights infringement, cultural discrimination); and (c) working conditions. Resilience factors: (a) social connections; (b) recognition and respect; (c) care recipient’s functional status; (d) relationships with family/friends; (e) working conditions; (f) professional experience; (g) leisure activities; and (h) work motivation (financial, altruistic).	Relational dynamics: satisfaction and types of relationships (migrant care workers–employer families and migrant care workers–older adults) Job satisfaction, abuse and neglect Well-being: migrant care workers (coping strategies and mental health)
**Lai and Fong (2020** **)**	Hong Kong	To examine how working conditions and workplace environments influence aggressive behavior from employers toward migrant care workers in home-based settings.	Quantitative approach using anonymous standardized questionnaires and face-to-face interviews. Dependent variables: Frequency of being scolded, neglected, or hit by employers in the past 12 months. Independent variables: (a) Workplace environment; (b) Working conditions for live-in domestic workers.	2,017 female migrant care workers. Mean age: 35.8 years. More than half (67.9%) were from the Philippines and 32.1% were from Indonesia. Most had been in Hong Kong for an average of 3.8 years.	Both the workplace environment (e.g., home size) and working conditions (e.g., types of individuals served) are linked to experiences of abuse by employers toward female domestic workers.	Job satisfaction, abuse, and neglect
**Liew et al. (2020** **)**	Singapore	To explore the spatial negotiation of daily interactions between migrant care workers and their elderly care recipients using Alfred Schütz’s intersubjective “tuning” framework.	Qualitative study 69 semi-structured in-depth interviews were held, followed by “go-along” interviews with 20 older Singaporean participants, tracking their routes and routines for 7 days using GPS devices.	69 Singaporean older adults; 35 migrant care workers, 24 of whom were dyad-pairs. The care recipients were mostly female, and the migrant care workers were all female.	Migrant care workers often experience more negative than positive interactions due to their unequal status. However, the care worker-employer relationship is viewed as a continuous process of adjustment, with both parties relying on each other—workers for migration success and employers for essential care.	Relational dynamics: types of relationships (migrant care workers–older adults)
**Mazuz (2013** **)**	Israel	To describe familial dyads between the Filipina migrant care workers and the Israeli aged patient to broaden the understanding of the varieties of bodily-based practices.	Qualitative study Observation and participant-observation study at the homes of thirty Israeli patients cared for by Filipino migrant care workers. The study is based on ethnographic research among Filipino migrant care workers in Israel.	30 Israeli patients cared for by Filipino migrant care workers. Most of the patients were Jewish-Israeli female citizens, widowed mothers aged 68 or more.	The family care relationship highlights how care practices are dynamic and complex, involving both mental and physical connections between people. The familial dyad then became a bonding connection created solely between the migrant care worker and the patient in which no one else, even the patient’s children, can participate. Older adult care in this context emerges as a dialogic process between two bodies and, therefore, it is constituted in terms of social and embodied relations.	Relational dynamics: types of relationships (migrant care workers–older adults)
**Mehta and Leng (2017** **)**	Singapore	To explore the dynamics of live-in foreign domestic workers and informal caregivers of older individuals.	Qualitative study Interviews with 30 adult children caregivers and 15 foreign domestic workers.	30 adult children caregivers. 15 live-in foreign domestic workers.	The main themes identified were: (a) challenges and dilemmas encountered by family caregivers; (b) fulfillment of filial responsibility in parental care; (c) balancing work and caregiving responsibilities: the role of migrant care workers; and (d) managing additional assistance: hiring migrant care workers and associated challenges.	Relational dynamics: Satisfaction with the relationships (migrant care workers–employer families) Well-being: migrant care workers (coping strategies) and employer families
**Munkejord et al. (2021a** **)**	Taiwan	To analyze the coping strategies of live-in migrant care workers from Indonesia and the Philippines.	Qualitative study Interviews with 13 live-in migrant care workers.	13 live- in migrant care workers; 12 Indonesian, 1 Filipino.	Two main coping strategies emerged: (a) “accepting destiny”—migrant care workers accepting their circumstances and viewing their role as fulfilling parental expectations of financial support (emotion-focused); and (b) “connecting with significant others”—the primary source of motivation for caregivers to persevere (behavioral).	Well-being: migrant care workers (coping strategies)
**Munkejord et al. (2021b** **)**	Taiwan	To explore the interrelationships and collaborative efforts between live-in caregivers and their employers	Qualitative study Interviews conducted as informal, semi-structured conversation to 10 employers and 10 live-in migrant care workers.	10 employers; 10 live-in migrant care workers (9 from Indonesia and 1 from Philippines).	“Unsupportive relationships”–live-in caregivers treated as servants; “Supportive relationships”–live-in caregivers treated as care workers; “Semi-supportive relationships”– live-in caregivers treated as carer-servants.	Relational dynamics: types of relationships (migrant care workers–employer families)
**Østbye et al. (2013** **)**	Singapore	To evaluate the moderating effect of instrumental support from migrant care workers on the relationship between four types of impairments in older adults (physical function, memory, behavioral, and mood) and caregiving outcomes among informal caregivers.	Quantitative Singapore survey on informal caregiving. Variables studied: CRA scale validated in Singapore; OARS; 24-item RMBPC; Instrumental Support from an migrant care worker.	1,181 older adult–informal caregiver dyads. Older adults: 83.4 years old, mostly women. Informal caregivers: 50–64 years old, female.	Instrumental support from migrant care workers, reported by 50% of older adults, moderated the relationship between impairments and caregiver outcomes. It buffered the effects of physical impairment on disrupted schedules and poor health, memory impairment on disrupted schedules, and health and financial difficulties, and behavioral impairment on lack of family support. It also improved caregiver esteem by buffering the negative effects of behavioral impairment and reducing the positive impact of mood impairment on esteem.	Well-being: employer families
**Septi-Mauludina et al. (2023** **)**	Taiwan	To explore the experiences of live-in foreign caregivers from Indonesia providing care to older stroke survivors and their families.	Qualitative study Semi structured interviews with 22 Indonesian care workers providing in-home care to older stroke survivors in Taiwan.	22 women, Muslim, mean age: 36 years, from 10 months to 10 years in Taiwan, and caregiving experience from 1 month to 9 years.	Six themes emerged: (a) migrant care workers’ background; (b) perception of stroke survivors’ health and functional status; (c) migrant care workers’ values and preferences; (d) consequences of caring for stroke survivors; (e) migrant care workers’ skills, abilities, and knowledge in caregiving; and (f) potential resources available to migrant care workers.	Relational dynamics: types of relationships (migrant care workers-employer families) Job satisfaction, abuse, and neglect Well-being: migrant care workers (mental health)
**Shinan-Altman and Ayalon (2019** **)**	Israel	To investigate perceived control among live-in and live-out home care workers and identify factors that contribute to perceived control in both caregiving contexts.	Quantitative study using questionnaires. Variables studied: Perceived control: 10-point Likert-type scale; Satisfaction with the relationship: 7-point Likert-type scale; Social relationships: Frequency of time spent with others (friends, family, etc.) in the past week; Burden: 12-item scale assessing burnout related to assistance with 6 ADLs (e.g., eating, dressing) and 6 IADLs.	338 live-in migrant care workers: mean age: 38.9 years, female. 185 local live-out home care workers; mean age: 53.1 years, female.	Both caregiver types reported high perceived control, with live-in workers reporting greater control. Key predictors included older age, better satisfaction with the care recipient and family relationships, and lower burnout. For live-in workers, satisfaction with social relationships was a stronger predictor of perceived control.	Well-being: migrant care workers (perceived control)
**Tam et al. (2018** **)**	Singapore	To analyze migrant care workers’ challenges and coping strategies and the support they need when caring for older adults.	Qualitative study Qualitative in-depth interviews with 25 foreign domestic workers.	25 migrant care workers (Indonesia, the Philippines, and Myanmar), caring for frail seniors, 19 caring for people with dementia. 5 healthcare staff.	Six subthemes were grouped into three main themes: social support from family members, coping strategies, and job satisfaction. Caregivers of seniors with dementia faced more challenges. Two family care models were identified: one in which migrant care workers handled most tasks, negatively affecting seniors’ well-being, and another in which family members and migrant care workers shared caregiving duties, resulting in better outcomes for older adults.	Relational dynamics: Satisfaction and types of relationships (migrant care workers-employer families) Well-being: migrant care workers (coping strategies)
**Teshuva et al. (2019** **)**	Israel	To investigate the quality and nature of care relationships between live-in migrant care workers and older individuals in Israel.	Mixed methods. A survey was conducted with 116 migrant care workers and 73 older care recipients using a 105-item questionnaire. The survey included both closed-ended and open-ended questions about the care recipients’ health and functional status, as well as the caregivers’ living and employment conditions, job satisfaction, and the quality of caregiving relationships. For care recipients, the same survey was used, excluding job satisfaction questions. Four questions assessed the quality of the interpersonal relationship: (a) feeling close, (b) getting along well, (c) understanding each other, and (d) an overall relationship rating. Additionally, three open-ended questions explored the meaning and elements of good-quality relationships.	116 migrant care workers from the Philippines, India, Sri-Lanka and Nepal. Mostly female with a mean age of 39 years. 73 older adults, mostly females, with a mean age of 86 years.	Both older adults and migrant care workers rated the four relationship items highly. Significant correlations were found between their responses, with the exception of one item (“get al.ng well”), which showed significant differences in t-test results. Qualitative data provided deeper insight into these ratings, revealing four key themes related to positive care relationships: emotional connection, reciprocity, effective communication, and meeting the older adult’s care needs.	Relational dynamics: satisfaction and types of relationships (migrant care workers–older adults)
**van Bochove and zur Kleinsmiede (2020)**	Netherlands	To explore how interactions within the care network influence the precarious work experiences of live-in migrant care workers.	Qualitative study In-depth interviews with migrant care workers in the Netherlands, as formal and informal caregivers, and the staff of intermediary organizations.	4 managers of intermediary organizations; 9 migrant care workers; 3 relatives of care recipients; 2 care coordinators; 1 district nurse.	In addition to care recipient conditions (physical, housing and social conditions), the interactions among stakeholders within the care network significantly influence the circumstances and experiences of migrant care worker work.	Relational dynamics: satisfaction and types of relationships (migrant care workers–older adults) Well-being: migrant care workers (coping strategies)
**Yeoh et al. (2023** **)**	Singapore	To examine how families allocate caregiving responsibilities between live-in migrant care workers and adult children, and to explore how these dynamics affect household relationships, particularly in the context of inter-generational caregiving alongside paid care.	Qualitative study In-depth interviews with 34 older adults cared for by migrant care workers and 35 migrant care workers employed to care for older adults.	Migrant care workers: 15 from Indonesia, 12 from Myanmar and 8 from the Philippines. Ages 20-40. Older adults: Aged 63-96, 22 females and 12 males.	Key themes included: (a) care arrangements; (b) daily challenges; (c) mobility patterns; (d) future aspirations; and (e) care relationships. Families hire migrant care workers for intimate care of older adults, fostering familial, trusting relationships, although challenges like surveillance or language barriers may emerge. Hiring an migrant care worker changes the family’s caregiving role.	Relational dynamics: types of relationships (employer families–migrant care workers)
**Yuan et al. (2022** **)**	Singapore	To determine whether engaging migrant care workers for dementia care moderates caregiver stress and to explore caregivers’ experiences in this context.	Mix methods Quantitative sub-study with 282 informal caregivers of people with dementia. ADL and IADL; RMBPC; ZBI; RSCSE; PAC. Qualitative sub-study in which 15 foreign domestic workers providing informal care were interviewed.	282 informal caregivers, mean age 55.7 years, mostly female (75.2%).	Engaging migrant care workers moderated depressive symptoms among dementia caregivers but did not affect caregiving burden, self-efficacy, or perceived positive aspects of caregiving. Qualitative findings revealed that the experience of engaging an migrant care worker is ambivalent, offering both support and challenges.	Relational dynamics: satisfaction with the relationships (migrant care workers–employer families) Well-being: employer families
**Zriker et al. (2024** **)**	Israel (Haredi society)	To provide deeper knowledge of the subjective meanings of integrating a migrant live-in care worker in the Haredi society, from the perspective of the family members (sons and daughters).	Qualitative study In-depth interviews with 15 family members.	11 women and 4 men primary caregivers of their elderly parents (≥62 years).	The Haredi family and the migrant live-in care workers must adapt to the cultural changes (i.e. cook in accordance with Jewish dietary law, engage in Shabbat-related customs, the use of technology and internet). Religious customs often serve as a link between live-in migrant care workers and families.	Relational dynamics: satisfaction with the relationships (migrant care workers–employer family)

*Note.* ADL = Activities of Daily Living; AMT = Abbreviated Mental Test; CRA = Multidimensional Caregiver Reaction Assessment; DJGLS = De Jong Gierveld Loneliness Scale; DRS = Dyadic Relationship Scale (DRS); GPS = Global Positioning System; HCSM = Home Care Satisfaction Measure; IADL = Instrumental Activities of Daily Living; JGLS = xxx; LSNS = Lubben Social Network Scale; NGO = Non-Governmental Organization; OARS = Older Americans Resources; PAC = Positive Aspects of Caregiving Scale; RMBPC = Revised Memory and Behavior Problems Checklist; RSCSE = Revised Scale for Caregiving Self-Efficacy; ZBI = Zarit Burden Interview.

To ensure methodological rigor and enhance the trustworthiness of the review process, investigator triangulation was employed. All articles were read in full by the first three authors, and they independently identified preliminary categories. These were then compared and discussed collectively; in cases of disagreement, they were addressed in discussion until consensus. This collaborative approach strengthened the reliability of the thematic analysis.

The analysis followed a thematic approach that combined both deductive and inductive strategies (Flick, 2018). Based on the research questions, initial codes were developed by consensus among the first three authors. The main categories identified included: relational dynamics, job satisfaction, abuse and neglect, and well-being. These were defined in a codebook to ensure consistency in application across researchers.

During the data extraction phase, the researchers also recorded which member(s) of the caregiving triad (migrant care workers, older adults, or employer families) the information referred to. Within the relational dynamics category, subthemes included quality, satisfaction, and types of relationships. Coding was performed independently by the first three authors, and any discrepancies or uncertainties were resolved through discussion. Finally, the last author (K.P.) reviewed the themes, codes, and the overall thematic analysis process to provide feedback for consideration by the original coders (Joffe & Yardley, 2004). The researchers therefore included a column indicating the theme(s) identified in the studies.

## Results

### Study selection

**Figure 1.**
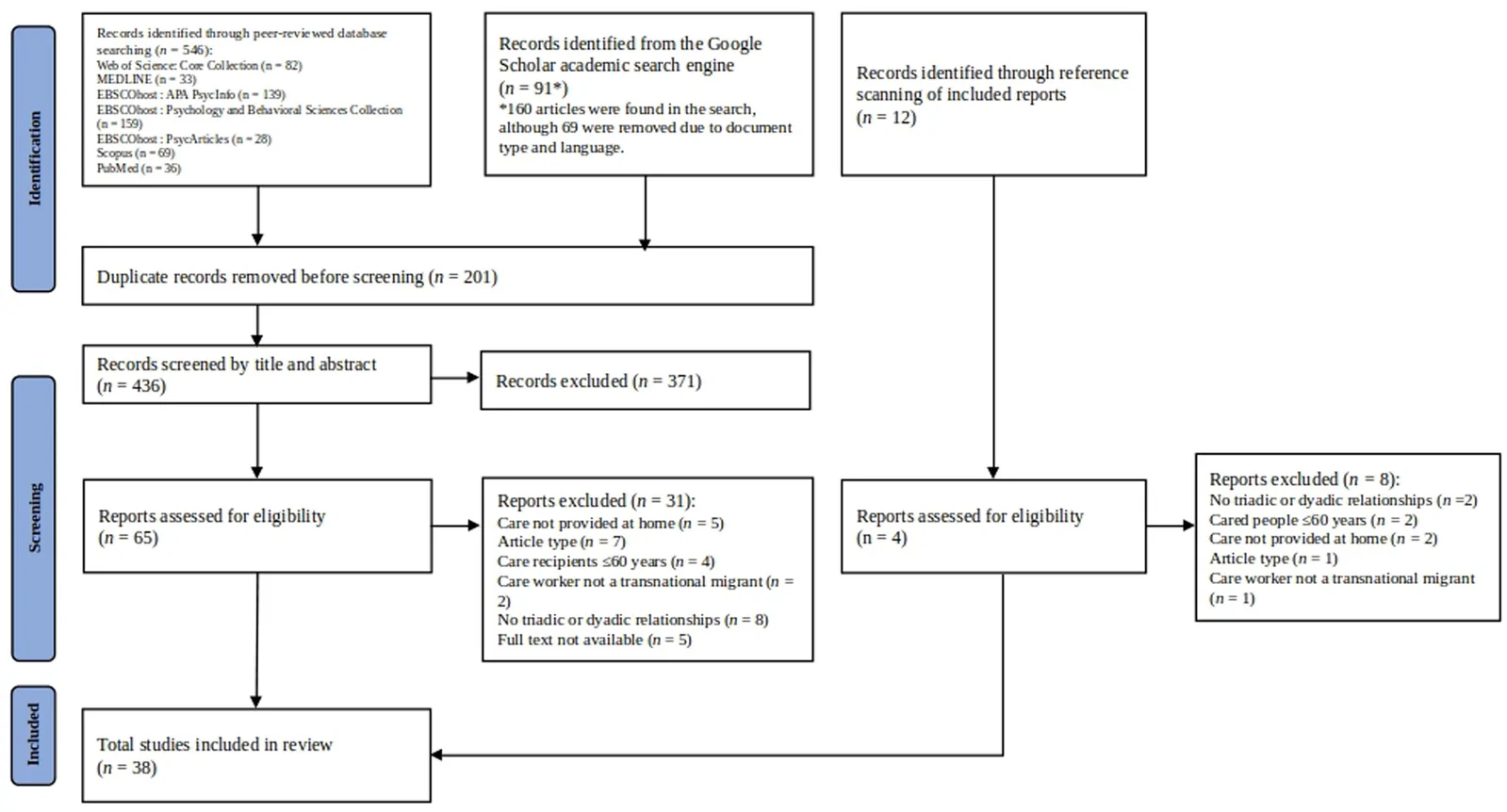
PRISMA flow diagram depicting the study selection process.

A total of 546 records were identified through databases: 82 in Web of Science core collection, 33 in Medline, 139 in EBSCOhost: APA PsycInfo, 159 in EBSCOhost: Psychology and Behavioral Sciences Collection, 28 in EBSCOhost: PsycArticles, 69 in Scopus, and 36 in PubMed (Figure 1). In addition, 91 records were obtained from Google Scholar and 201 duplicates were removed before screening. First, the titles and abstracts of 436 records were reviewed, resulting in the exclusion of 371. Inter-rater reliability for the first phase was κ = .830. Second, full-text scans inter-rater reliability was κ = .896, and were conducted of 65 eligible studies, with 34 being included. Reference list scanning yielded 12 eligible studies, of which 4 were included, resulting in a final collection of 38 studies.

### Characteristics of the included studies

In total, 38 studies met the inclusion criteria for this scoping review. A quantitative methodology was employed in 15 studies, while another 19 studies utilized qualitative methods, and 4 studies adopted a mixed-method approach. The perspective of migrant care workers was examined in 14 studies (Cohen-Mansfield & Golander, 2021; Goldzweig et al., 2015; Green & Ayalon, 2017, 2018; Heng et al., 2019; Ho et al., 2018, 2019; Kaur-Gill & Dutta, 2021; Kriegsmann-Rabe et al., 2023; Lai & Fong, 2020; Munkejord et al., 2021a; Septi-Mauludina et al., 2023; Shinan-Altman & Ayalon, 2019; Tam et al., 2018), 4 studies analyzed the triadic perspective (Ayalon & Roziner, 2016; Ayalon, 2016; Green & Ayalon, 2015; Iecovich, 2016), and only 4 studies examined the perspective of employer families (Basnyat & Chang, 2017; Grenz & von Kutzleben, 2024; Yuan et al., 2022; Zriker et al., 2024). Finally, a total of 11 studies analyzed the perspective of dyadic relationships. Specifically, older adults and families ([Bibr gnaf300-B68][Bibr gnaf300-B8]; ), older adults and migrant care workers (Liew et al., 2020; Mazuz, 2013; Teshuva et al., 2019; Yeoh et al., 2023), and families and care workers (Ayalon et al., 2025; Cohen-Mansfield & Golander, 2023; Mehta & Leng, 2017; Munkejord et al., 2021b; Mehta & Leng, 2017; van Bochove & Zur Kleinsmiede, 2020). Finally, the particular perspective of older adults was explored in 3 studies (Ho et al., 2022, 2023; Hung et al., 2024), and 2 studies (Cohen-Mansfield & Golander, 2022; Hoens & Smetcoren, 2023) explored family or older adults and migrant care workers perspective.

In terms of geographical location, 16 studies were conducted in Israel (Ayalon & Roziner, 2016; Ayalon et al., 2025; Ayalon, 2014, 2016; Cohen-Mansfield & Golander, 2021, 2022, 2023; Goldzweig et al., 2015; Green & Ayalon, 2015, 2017, 2018; Iecovich, 2016; Mazuz, 2013; Shinan-Altman & Ayalon, 2019; Teshuva et al., 2019; Zriker et al., 2024), 9 in Singapore (Basnyat & Chang, 2017; Heng et al., 2019; Kaur-Gill & Dutta, 2021; Liew et al., 2020; Mehta & Leng, 2017; Østbye et al., 2013; Tam et al., 2018; Yeoh et al., 2023; Yuan et al., 2022), 5 in Hong Kong (Ho et al., 2018, 2019, 2022; Hung et al., 2024; Lai & Fong, 2020), 3 in Taiwan (Munkejord et al., 2021a, 2021b; Septi-Mauludina et al., 2023), 2 in Germany (Grenz & von Kutzleben, 2024; Kriegsmann-Rabe et al., 2023), 1 in the Netherlands (van Bochove & Zur Kleinsmiede, 2020), 1 in Hong Kong and the United Kingdom (Ho et al., 2023), and 1 in Belgium (Hoens & Smetcoren, 2023).

### Caregiving experiences and relationships between the three parties

In this section, we present the findings of the thematic analysis carried out as part of the scoping review. The findings are grouped under the following themes: (a) relational dynamics (satisfaction, quality, and types of relationship); (b) job satisfaction, abuse and neglect; and (c) well-being.

#### Relational dynamics (satisfaction, quality and types of relationship)

In this section, the researchers describe the relational dynamics between the three parties identified in the studies analyzed. To the extent that results are available, they will be described in the following order: migrant care worker and employer family, migrant care worker and care recipient, and, if applicable, the triadic relationship.

##### Satisfaction and quality of the relationships

Several studies aimed to analyze satisfaction with the relationships between migrant care workers and employer families. Families frequently choose live-in migrant care due to limited formal services and desire for continuous, person-centered support that alleviates caregiver burden and enables older adults to remain at home (Hoens & Smetcoren, 2023; Zriker et al., 2024). However, this decision often stems from a lack of better options and concerns about nursing homes, requiring families to adapt to challenges of incorporating a live-in carer (Grenz & von Kutzleben, 2024).

From the family perspective, experiences are often ambivalent (Yuan et al., 2022). Families value support with daily caregiving tasks and emotional aspects, as well as improved quality of life enabling older adults to remain at home. Conversely, families face challenges finding suitable care workers, managing retention, navigating absent legal regulations, and addressing issues with caregiving skills, appropriate attitudes, and workers’ personal and health needs. Some families reported problems with inexperienced migrants and did not rate support for people with dementia highly (Mehta & Leng, 2017).

Cultural differences pose challenges. Language barriers and limited dementia knowledge force families to act as cultural and communicative intermediaries between people with dementia and migrant care workers (Ayalon et al., 2025). In Haredi society, for example, integrating migrant care workers presented unique challenges due to conflicts with Jewish law, including dietary rules, technology use, and dress codes. However, religion can also foster closer relationships, providing common ground despite differences (Zriker et al., 2024). The combination of unmet expectations, limited dementia-specific competencies, and families’ demands for constant availability can generate conflicts straining care arrangements (Grenz & von Kutzleben, 2024).

Some migrant care workers report strain and fear in their relationships with employer families, although others describe positive interactions, including affirmation, gifts, training opportunities, adequate rest, and shared caregiving (Tam et al., 2018). Kriegsmann-Rabe et al. (2023) found that many migrant care workers reported respectful and grateful interactions with the families of care recipients. Ayalon et al. (2025) found that migrant care workers emphasized maintaining positive relationships with families, characterized by trust, respect, and protection when caring for people with dementia, as essential for addressing caregiving challenges. They highlighted the need to acquire knowledge about the older person, recognize family members as mediators, and attend to their own well-being. As an exception, migrant care workers caring for older adults with cancer felt less attached to families compared with migrant care workers caring for those with other chronic illnesses (Goldzweig et al., 2015).

Regarding migrant care worker–care recipient relationships, studies identified two main challenges: language barriers and issues around caring for people with dementia. Language barriers caused misunderstandings (Heng et al., 2019; Teshuva et al., 2019) and caring for people with dementia was perceived as uniquely difficult. Verbal and physical aggression from people with dementia caused relationship strain, and disinhibition in public generated fear about leaving the house, resulting in worker isolation (Tam et al., 2018). Migrant care workers perceived repetitive questions and disrupted sleep–wake cycles as challenges but attributed behavioral problems to the disease, emphasizing that caring with love, patience, and repetitive communication was essential (Ayalon et al., 2025). However, other studies (Kriegsmann-Rabe et al., 2023; Mehta & Leng, 2017; Teshuva et al., 2019) found that strained migrant care worker–people with dementia relationships led to conflicts with employer families.

Regarding quality of relationships between migrant care workers and employer families, Ayalon (2014) found it was conveyed more through satisfaction with work performed than interpersonal dynamics. Cohen-Mansfield and Golander (2023) found significant correlation between quality ratings by migrant care workers and families, though families rated relationships significantly more positively. In their qualitative data, migrant care workers cited mistrust as a main reason for lower ratings.

Migrant care workers often relied on employers for legal, medical, and bureaucratic support due to immigration status, while families depended on care workers as finding replacements was difficult and time-consuming. Mutual trust, respect, appreciation, empathy, support, and conflict management emerged as critical determinants of relationship quality. Similarly, Ayalon et al. (2025) noted employer families emphasized good communication and maintained positive relationships with care workers, including additional compensation and flexible arrangements, to ensure worker satisfaction and high-quality care for people with dementia.

Ayalon et al. (2025) also demonstrated that mutual relationships between migrant care workers and employer families were essential for adequate people with dementia care. Grenz & von Kutzleben (2024) emphasized that care quality also depended on workers’ knowledge of care recipients’ needs and preferences, linguistic and communicative competencies, cultural fit, and promotion of social participation. Cohen-Mansfield and Golander (2022) found that the quality of the relationship was the only significant predictor of the caretaking dimension of care. Overall, positive relationships marked by mutual trust and engagement directly enhanced both caretaking and person-centered care, whereas employment conditions indirectly affected care quality through their impact on workers’ perceptions.

Regarding the quality of relationships between migrant care workers and care recipients, Teshuva et al. (2019) found that both groups rated it highly. Most participants reported feeling close, understanding each other, and getting along well, viewing care quality as central to relationship success. Cohen-Mansfield & Golander (2022) found that care quality was most strongly predicted by relationship quality between migrant care workers and older care recipients, as well as between migrant care workers and relatives. Relationship quality also improved significantly when care recipients were female and needed more assistance with daily activities.

However, migrant care workers rated relationship quality more positively than older adults. Ho et al. (2023) found poor relationship quality with migrant care workers correlated with greater social and overall loneliness, although not emotional loneliness, whereas better relationships linked to lower loneliness. Care recipients’ medical conditions significantly influenced migrant care workers’ jobs, often determining their ability to take time off (van Bochove & Zur Kleinsmiede, 2020).

Ayalon and Roziner (2016) analyzed the caregiving triad, focusing on satisfaction variability and reciprocity. They found that migrant care workers were the main source of variability, although they played a passive role, as outcomes were more influenced by family members’ and older adults’ satisfaction levels than by those of workers. Satisfaction within the triad was reciprocal: Higher satisfaction in one member often led to higher satisfaction in another. However, unexpectedly, as family members’ satisfaction increased, migrant care workers’ satisfaction tended to decrease, and vice versa.

Cohen-Mansfield and Golander (2023) found both family caregivers and migrant care workers perceived that relationship quality between the care worker and older person influenced the migrant care worker–family caregiver relationship. Similarly, the family–older person relationship was perceived to impact the migrant care worker–family caregiver relationship. Iecovich (2016) examined ambivalent dyadic relationships between triad members and their impact on care recipients’ loneliness, finding moderate ambivalence levels, with family caregivers reporting higher ambivalence than migrant care workers. Higher ambivalence in these relationships was linked to increased loneliness among care recipients.

##### Types of relationship

Regarding migrant care worker–employer family relationships, different types emerged. Munkejord et al. (2021b) identified three types: *unsupportive relationships*, where care workers were treated as servants with round-the-clock duties and restricted family contact (employers justified this claiming good pay); *supportive relationships* (mainly in middle-class families), where workers were treated respectfully with focus on caregiving over household chores, allowed limited free time and digital family contact; and *semi-supportive relationships*, combining caregiving with household duties, with employers acknowledging inability to provide more support due to financial or time constraints.

Similarly, Tam et al. (2018) described worker-centered dyna­mics where migrant care workers handled most duties, increasing stress and negatively affecting care recipients’ well-being, versus team-based dynamics where shared responsibilities reduced migrant care worker stress and improved care recipients’ well-being. Yeoh et al. (2023) highlighted power imbalances, with migrant care workers facing limited support, reprimands for taking initiative, and employer surveillance, reflecting underlying tensions and mistrust. However, Cohen-Mansfield and Golander (2023) found that while some migrant care workers and employed families described the relationship as “feeling like family,” others reported feelings of distance or uncertainty which can be conceptualized along two axes: a qualitative axis ranging from kinship-like connections to formal employment relationships and ambiguous forms in between, and a quantitative axis reflecting the intensity of the relationship.

Ho et al. (2018) analyzed the relationship from migrant care workers’ perspective, finding migrant care workers were employed to perform burdensome and “dirty” tasks, simultaneously satisfying their desires to earn money and pursue career aspirations. However, this instrumental framing reduced their autonomy and self-worth, as their identities became commodified in service-for-money exchanges. This situation was often perceived as unchangeable, and work meaning fluctuated between being treated as commodities or maids versus valued as companions or friends, depending on trust and respect within caregiving relationships.

In terms of relationships between migrant care workers and care recipients, the dynamics that emerge between the two members of this dyad are very important for a successful and intimate relationship. Liew et al. (2020) described this dynamic as a relational process requiring ongoing “tuning,” as care relationships are co-constructed through everyday interactions. A growing body of research highlights the key components of positive care relationships. Teshuva et al. (2019) identified four key themes in positive care relationships: emotional connections (e.g., family-like bonds), reciprocity (mutual respect and appreciation), effective communication (active listening and open dialogue), and meeting the older person’s care needs. Kriegsmann-Rabe et al., (2023) found that emotional bonds (friend- or family-like relationships) allowed for mutual learning, shared enjoyment, and support.

Building on this concept, Hung et al. (2024) emphasized the dynamic and evolving nature of these relationships, shaped by daily interactions and contextual factors. Their study identified four types of relational interaction: (a) approximate family companionship and care, (b) trust and sharing initiate emotional attachment, (c) merely employer–employee relations avoid interaction, and (d) behaviors influencing communication and relationships (mutual respect, loyalty, honesty) foster strong relationships with migrant care workers.

In related work, Ho et al. (2018) introduced the notion of “destined reciprocity,” wherein moments of mutual trust and recognition transcend the instrumental nature of paid care, allowing both parties to experience authenticity and shared meaning in the caregiving relationship. As a consequence, migrant care workers prioritize the “destined reciprocity” over their monetary or financial rewards. Similarly, Mazuz (2013) observed that the intimate nature of care practices (e.g., physical proximity, attentive touch, and gestures involving the face and hands) can foster family-like bonds between migrant care workers and older adults, often transforming the relationship into one resembling a mother–daughter dynamic.

This unique bond, formed through everyday care routines, is exclusive to the caregiver–care recipient dyad and excludes even the older person’s relatives. Such embodied care becomes particularly important when language or cultural barriers limit verbal interaction. Consistent with these findings, Hoens and Smetcoren (2023) found that nonverbal forms of communication, such as attentiveness, patience, friendliness, and compassion, were crucial in the relationship. However, several older adults, particularly partners, reported a loss of privacy and the need to adjust to the constant presence of a live-in carer and the shifting dynamics of power and control. Over time, most older adults and their partners found a balance in the relationship, with many eventually coming to view the live-in migrant care workers as family members.

#### Job satisfaction, abuse, and neglect

Job satisfaction, rights, abuse, and neglect among migrant care workers emerged as major themes in the research literature. Green and Ayalon (2015) found persistent abuse of workers, despite moderate rights awareness, with older adults being least informed. Employment rights, such as sick leave and vacation, were often violated. Recent studies (Green & Ayalon, 2017, 2018) reported high rates of emotional and physical abuse, limited vacation days, lack of contracts, and inadequate living conditions.

Hoens and Smetcoren (2023) found that clear role descriptions were often absent when older adults hired migrant care workers directly, with most cases having only verbal rather than written contracts. Older adults paid carers directly, with monthly wages ranging from €750 to €1500 for 24-hr care with one day off weekly. Stay duration varied widely from 2 months to over 5 years. Due to vague employment terms, some migrant care workers lacked fixed days off. When migrant care workers were hired by agencies, employment conditions improved, although this created a financial burden for older adults. Live-in workers were more vulnerable than live-out workers, facing 24-hr care demands, isolation, and interrupted rest (Kriegsmann-Rabe et al., 2023). Other stressors included cultural racism, financial fraud, and workplace abuse, exacerbated by power imbalances in domestic settings (Kaur-Gill & Dutta, 2021; Lai & Fong, 2020). Tasks often extended beyond caregiving to include nursing duties, emotional support, and household responsibilities (Heng et al., 2019).

Cohen-Mansfield and Golander (2021) found job satisfaction depended on work relationships and employment conditions, not on care recipients’ functional status. Many migrant care workers reported dissatisfaction with low pay, lack of privacy, insufficient time off, and unmet rights (Septi-Mauludina et al., 2023), whereas creativity and resourcefulness in caregiving improved satisfaction (Tam et al., 2018), particularly among untrained workers caring for people with dementia.

Predictors of worker abuse included care recipients’ cognitive impairments, longer employment, and household size, with male care workers more susceptible than females (Green & Ayalon, 2017). Financial constraints trapped workers in abusive environments. Regarding older adult neglect, Ayalon (2016) found 31.5% of older adults, 18% of migrant care workers, and 32.3% of family members reported at least one type of neglect. In Israel, live-in care reduced family burden and neglect compared to native live-out caregivers; however, home care worker burden was linked to higher neglect rates.

#### Well-being

In this section, the results linked to the well-being respectively of migrant care workers, care recipients, and family members are focused.

##### Migrant care workers

Several studies analyzed migrant care workers’ well-being, emotion management, coping strategies, perceived control, mental health, and burnout.

###### Emotion management

Migrant care workers exhibit moral habitus—moral judgment of oneself and others developed through embodied emotions. They employ this judgment in managing relationships through three approaches: (a) developing and expressing protective feelings and love, often regulating emotions to meet sociocultural workplace demands, even displaying fake emotions; (b) experiencing genuine emotions such as compassion or distress in the older person’s best interests; and (c) demonstrating emotions, fake or genuine, to ensure good stewardship (Ho et al., 2019).

###### Coping strategies

Munkejord et al. (2021a) identified two coping strategies: (a) “accepting destiny” (emotion-focused), focusing on supporting family in their home country, viewing caregiving as earning means; and (b) “connecting to significant others” (behavioral), finding motivation through relationships with care recipients, employer families, and their own families.

Social mobility, such as interacting with other migrant care workers or using mobile phones, provided emotional support (Heng et al., 2019; Kaur-Gill & Dutta, 2021; Mehta & Leng, 2017; Tam et al., 2018; van Bochove & Zur Kleinsmiede, 2020). Other strategies included adequate breaks, although some workers close to care recipients said they did not require time off. Socializing, praying, and hobbies such as singing or dancing were helpful (Heng et al., 2019; Mehta & Leng, 2017).

High self-determination and good relationships contributed to migrant care workers’ happiness and resilience (Kriegsmann-Rabe et al., 2023). How migrant care workers perceived their role greatly impacted well-being and stress management. Family-like relationships with care recipients’ relatives or friends served as crucial support networks. Long-term workers in the Netherlands built non-client social networks, whereas those traveling back and forth focused solely on caregiving with limited opportunities for such connections (van Bochove & Zur Kleinsmiede, 2020).

###### Perceived control

Shinan-Altman and Ayalon (2019) studied perceived control among 338 live-in migrant care workers and 185 local live-out home care workers in Israel. Both groups reported high perceived control, with live-in migrant care workers reporting more control but also higher burnout. Age, satisfaction with care recipient and family relationships, and lower burnout predicted perceived control, with satisfaction with social relationships being a stronger predictor for live-in workers.

###### Mental health

Septi-Mauludina et al. (2023) found stroke survivors’ migrant care workers faced physical and psychological stressors. Limited caregiving knowledge led to frustration, helplessness, and anxiety, while round-the-clock care worsened psychological stress, leading to depression. Conflicts with care recipients or families and abusive work environments contributed to mental health issues. Cohen-Mansfield and Golander (2021) found work-related factors, such as care recipients’ functional status and workers’ relationships with them, directly impacted burnout, while employment conditions influenced it indirectly. Migrant care workers to cancer patients reported significantly higher distress and negative caregiving impact than caregivers of patients with other chronic illnesses (Goldzweig et al., 2015).

Ayalon (2016) found more informal family assistance was linked to lower migrant care worker burden. Migrant care workers often faced choosing between enduring poor conditions or leaving, both affecting well-being (Kaur-Gill & Dutta, 2021). In contrast, Kriegsmann-Rabe et al., 2023 found most live-in migrant care workers, regardless of legal status, reported good job satisfaction, with social relationships being a key factor.

##### Care recipients

Regarding care recipients’ well-being, several studies analyzed loneliness. Ho et al. (2022) reported 35.3% of care recipients experienced moderate-to-severe loneliness. Co-residence with a migrant care worker reduced overall loneliness and emotional loneliness but had no impact on social loneliness. Hung et al. (2024) found 44% of older adults felt lonely with social isolation and reduced social engagement (50.7%). Although older adults reported poor mutuality with live-in migrant care workers in surveys, interviews revealed migrant care workers contributed to reducing loneliness and social isolation by fostering positive relationships. Iecovich (2016) found health, economic status, and dyadic ambivalence with family caregivers explained 26% of variance in loneliness among care recipients, but their migrant care worker relationship had no impact.

##### Employer families

Yuan et al. (2022) found migrant care workers support reduced depression symptoms in families but did not affect caregiving burden, self-efficacy, or perceived positive aspects. Conversely, Ayalon (2016) and Østbye et al. (2013) found migrant care workers assistance improved caregiver burden, particularly as care recipients’ impairments increased. Basnyat and Chang (2017) reported migrant care workers helped reduce burnout and financial strain, fostering mutual support relationships. However, family caregivers faced stress related to finances, physical and emotional strain, routine adaptation, and lack of appreciation from care recipients (Mehta & Leng, 2017).

## Discussion

This is the first scoping review to analyze the extent and nature of the caregiving experiences and relationships among migrant home care workers, older adult care recipients, and employer families. The review examined 38 studies exploring relational dynamics, job satisfaction, abuse and neglect, and the well-being of all parties involved in the care triad. Several overarching themes from this analysis are particularly noteworthy.

### Relational dynamics

A major finding highlights the fact that relational care dynamics between migrant care workers, family caregivers and older care recipients are highly complex. These multifaceted relationships are influenced by cultural, social and economic factors that affect the well-being of all parties involved, as well as the quality of the care provided (Ayalon et al., 2025; Cohen-Mansfield & Golander, 2023; Salami & Meherali, 2018). Satisfaction with relationships varies widely across the different parties. However, interactions characterized by trust, respect, mutual respect and acknowledgment positively impact the caregiving outcomes (Kriegsmann-Rabe et al., 2023; Tam et al., 2018). Previous research highlighted the critical importance of these features in the live-in migrant care worker–employer relationships (Salami & Meherali, 2018).

Communication and cultural barriers have been identified as significant challenges associated with caregiving experiences, as reported by all the parties involved (Ayalon et al., 2025; Teshuva et al., 2019). In particular, migrant care workers reported tensions associated with caring for people with dementia (Tam et al., 2018) and older adults with cancer (Goldzweig et al., 2015). Several studies reported ways in which language and communication barriers can negatively impact care quality, in turn limiting effective caregiving and understanding (Xu, 2008) and influencing care relationships (Johnstone & Kanitsaki, 2009). These factors shape both the practical and emotional dynamics of caregiving.

Regarding types of relationships, supportive relationships were associated with better outcomes for all parties, whereas their unsupportive counterparts exacerbate power imbalances (Tam et al., 2018; Yeoh et al., 2023). These imbalances were strongly influenced by structural inequalities including gender, class and race, which give rise to exploitation, unsafe working conditions and a lack of acknowledgment (Cohen-Mansfield & Golander, 2023; Frantz, 2008). Other studies have also reported similar results. For instance, Salami and Meherali (2018) found a professional employer-employee relationship characterized by clearly defined boundaries and the absence of emotional attachment; a familial relationship, which often begins with a friendship and evolves into employers perceiving live-in caregivers as part of the family; and a hybrid relationship, which lies between the professional and familial models, incorporating elements of both.

In terms of the type of relationship established between migrant care workers and their older care recipients, this scoping review indicates that such relationships are co-constructed through positive daily interactions (e.g., emotional connections, reciprocity) (Hung et al., 2024; Liew et al., 2020; Teshuva et al., 2019). In addition, non-verbal communication creates a more family-like relationships between migrant care workers and older adults (Mazuz, 2013) and the studies included in this review highlighted the importance of family-like bonds in fostering resilience, mutual learning, and shared enjoyment, emphasizing that quality of care is central to the success of these relationships (Cohen-Mansfield & Golander, 2023; Heng et al., 2019; Kriegsmann-Rabe et al., 2023; Septi-Mauludina et al., 2023; Tam et al., 2018).

Previous research has shown that care encompasses not only the physical tasks involved in caregiving, but also the relational dynamics between the caregiver and the care recipient, as well as the interplay between the practical and emotional dimensions of the caregiving process (Himmelweit, 1999). Nolan et al. (2004) extend this perspective by advocating for a shift from individualistic, person-centered care to relationship-centered care, emphasizing the interdependence between care workers and care recipients and the crucial role played by interpersonal interactions in establishing a foundation for effective care (Nolan, 1997, cited in Walsh & Shutes, 2013). The authors claim that older adult care workers in home settings faced vulnerability due to “blurred emotional boundaries” and the increased emotional demands placed on them by care recipients (Lovelock & Martin, 2016).

Some studies have drawn attention to the existence of a structural relationship of dependence between older adults, their family members and migrant care workers, which is based on economic, physical, and emotional aspects. Similarly to Cohen-Mansfield & Golander (2023), this study highlighted an interdependence between employer families and migrant care workers. Employers often frame live-in caregivers as “part of the family,” expecting emotional labor and traits like cheerfulness and positivity. This dynamic, amplified by shared living spaces, blurs the line between work and home. Although employers view this arrangement as supportive, care workers are more aware of its exploitative potential, as familial rhetoric often masks power imbalances and unpaid labor demands (Bakan & Stasiulis, 1997; Constable, 2007; Parreñas, 2001). These ambiguous relationships highlight the need to establish clear labor standards that guarantee fair and equitable work environments.

### Job satisfaction, abuse, and neglect

This scoping review highlights that job satisfaction among migrant care workers is more strongly influenced by work relationships and employment conditions than by the care recipient’s functional status (Cohen-Mansfield & Golander, 2021). Contributing factors to job dissatisfaction include low wages, lack of privacy, long hours, and unfulfilled rights (Green & Ayalon, 2015; Septi-Mauludina et al., 2023). High rates of emotional and physical abuse, poor living conditions, and absence of formal contracts were also noted, particularly among live-in workers facing continuous care demands, isolation, and disrupted rest (Green & Ayalon, 2017, 2018; Kriegsmann-Rabe et al., 2023). Power imbalances and cultural racism further limited migrant care workers’ ability to seek support or advocate for fair conditions (Kaur-Gill & Dutta, 2021; Lai & Fong, 2020). Similar results were found by Cheung et al. (2019), who analyzed the prevalence of abuse and depression Filipino domestic workers in Hong Kong. The results revealed high levels of emotional (58%) and physical abuse (18%), as well as a significant correlation between abuse and mental health, as 42% of those who reported having been abused also reported moderate to severe symptoms of depression. Co-residence with employers, language barriers, and financial dependence were key vulnerability factors, especially in live-in roles.

### The well-being of all parties

This scoping review found that the well-being of all parties involved was influenced by a combination of work-related, emotional, and social factors. Live-in migrant care workers report high stress levels due to long working hours, lack of privacy, and power imbalances. Emotional and physical abuse also affects their well-being, particularly when their labor rights are violated (Green & Ayalon, 2017; Kriegsmann-Rabe et al., 2023). Previous research shows how the psychological well-being of migrant care workers is closely linked to the quality of their work and family relationships (Aguirre & Ranea, 2020; Gallart, 2007). Factors such as social isolation, discrimination, and migratory grief negatively impact their mental health, leading to stress, anxiety, and depression. Providing training, work experience, and language classes, strengthening social support networks, promoting clear communications, and facilitating access to mental health services may help mitigate these negative effects (Bai et al., 2013; Ha et al., 2018; Monguí-Monsalve et al., 2022).

For their part, employer families value the support provided by migrant care workers and hiring them reduces symptoms of depression (Yuan et al., 2022). However, they also face difficulties adapting to the new family dynamics and their newfound dependence on caregivers from outside the family. Moreover, the mental health of employer families is often affected by stress and emotional burden, particularly when the care recipient suffers from cognitive impairment (Mehta & Leng, 2017). This situation in turn may lead to feelings of exhaustion, anxiety and, in some cases, depression (Østbye et al., 2013). These findings are confirmed by the results reported by Salami et al. (2017), who observed how relationships characterized by mutual support between migrant care workers and employer families helped alleviate families’ emotional burden. Nevertheless, conflicts between the two parties may intensify stress and anxiety, a circumstance that may affect families’ perceived self-efficacy as caregivers, particularly when the situation is exacerbated by the care recipient’s behavioral problems or the migrant care worker’s lack of experience (Basnyat & Chang, 2017; Liang, 2014).

Finally, positive relationships between migrant care workers and older care recipients were found to significantly decrease older adults’ levels of loneliness and social isolation, thereby enhancing their general well-being and increasing social participation. These findings are confirmed by a study carried out by Baldassar et al. (2017), who observed that respectful care relationships based on trust are essential to improving the well-being of all parties involved, whereas cultural barriers and social isolation may have a negative impact.

## Limitations

One of the major limitations of this scoping review was the methodological diversity of the studies included. The review incorporated quantitative, qualitative, and mixed-method approaches, which made it sometimes challenging to synthesize and compare findings. This diversity in study design also posed difficulties in conducting a thematic analysis, as each methodology provided different types of data, complicating the process of drawing consistent and generalized conclusions.

The generalization of the results of this scoping review is limited due to a cluster of studies by Ayalon et al. and a concentration of research from Israel and Singapore. A sensitivity analysis indicated that key conclusions are supported by a broader range of studies beyond this cluster. Nevertheless, the predominance of certain authors and regions should be considered when interpreting the results (see online supplementary material, Supplementary Material 2). The different cultural and geographical contexts where the studies were conducted may influence caregiving practices, family dynamics, and the experiences of migrant care workers.

Moreover, caregiving dynamics are highly context-dependent, and are influenced by local cultural norms, immigration policies, and caregiving practices. The findings of this scoping review must be interpreted within the broader context of the specific migration regimes, labor protection frameworks, and long-term care systems in which the caregiving arrangements occur. Further, findings on abuse prevalence, working conditions, and migrant care worker well-being are likely substantially influenced by the robustness of existing labor protection legislation and the effectiveness of enforcement mechanisms available within each country, requiring attention to these structural factors when interpreting and applying the findings across different policy environments.

Furthermore, many studies relied on self-reported data from migrant care workers, employer families, and older adults, introducing potential recall or social desirability bias, which may affect the reliability of findings, especially regarding sensitive issues such as abuse and exploitation. Moreover, the number of studies included in the scoping review varied in accordance with the perspective taken (migrant care workers, the older adult, the employer family, or a combination of these) a circumstance that further complicated the synthesis of results due to differing priorities and experiences across these groups.

Finally, despite a comprehensive search strategy, the review may have excluded relevant studies published in languages other than English or Spanish, potentially overlooking important perspectives from non-English-speaking regions with significant migrant caregiving populations.

## Conclusions and implications for research and policy

In conclusion, this scoping review revealed that caregiving dynamics between migrant care workers, older adults, and employer families are deeply influenced by cultural, structural, and interpersonal factors. Positive relationships improve the well-being of all parties involved, although challenges such as abuse, exploitation, and poor working conditions persist, especially for live-in caregivers. Strengthening these dynamics, improving working conditions, and effective legal and institutional support mechanisms for migrant care workers are critical to enhancing the quality of the care provided and the well-being of all those involved in the caregiving process.

Triadic relationships reveal tensions stemming from power imbalances and misaligned expectations. These findings emphasize the urgent need for more effective regulatory frameworks, enhanced psychosocial support, and the development of cultural humility training programs for all parties involved in the caregiving process. Cultural humility, which emphasizes ongoing self-reflection, recognition of power dynamics, and the necessity for mutual learning among all participants, represents a more appropriate framework than cultural competency approaches (Lekas et al., 2020). The incorporation of cultural humility training for migrant care workers, older care recipients, and employer families may help reduce cultural barriers, foster collaborative relationships based on mutual respect, and address the power dynamics inherent in these caregiving arrangements that were identified throughout this review.

In addition, structured feedback mechanisms, open communication channels, and language training are essential to aligning expectations and improving the quality of the care provided. In particular, language training plays a crucial role in dyadic and triadic caregiving relationships, where effective communication is key to fostering trust, understanding, and coordination among migrant care workers, care recipients, and families. In addition, comprehensive language training programs should be implemented for all parties involved in caregiving relationships. Such efforts should include providing host country language instruction for migrant care workers to improve their communication abilities, as well as basic language training for employer families and care recipients in workers’ native languages where feasible. Such bidirectional language support can help reduce communication barriers, minimize misunderstandings, and demonstrate mutual respect across cultural and linguistic differences.

Moreover, the findings of this review highlight that migrant care workers, particularly women from LMICs providing live-in care, face heightened risks of various forms of violence and exploitation due to their social isolation and precarious legal status (Office of the High Commissioner for Human Rights, OHCHR, 2024). Although increasing awareness of caregivers’ rights represents an important step, addressing these systemic vulnerabilities requires more robust institutional and legal support mechanisms, including regular monitoring systems through nongovernmental organizations or state-funded programs, accessible reporting mechanisms without fear of deportation or employment termination, and the establishment of independent oversight bodies with enforceable labor standards. The development of emergency shelter services, legal aid programs, and clear pathways for workers to seek redress are essential components of a comprehensive approach to addressing the power imbalances and structural vulnerabilities identified throughout this review.

Finally, providing accessible psychosocial support resources, such as counseling services and peer support networks, and helping migrant care workers manage stress and cope with isolation and emotional challenges are crucial to improving their mental health. Employer families and older adults should also have access to support services to reduce caregiving-related burden and feelings of loneliness, respectively.

This scoping review reveals several important areas requiring further investigation. First, longitudinal studies are needed to examine how caregiving relationships evolve over time and identify factors that contribute to relationship sustainability and quality. Second, there is a notable lack of research examining the triadic perspective comprehensively, with only four studies in this review adopting this approach. Moreover, few studies explore the experiences of older adults themselves, whose voices remain underrepresented in the literature. Future research should prioritize designs that simultaneously capture the perspectives of all three parties involved in the caregiving arrangement, including that of the care recipient.

Third, intervention studies are needed to evaluate the effectiveness of a range of possible programs, such as cultural humility training programs, conflict resolution strategies, and psychosocial support mechanisms identified as potentially beneficial in this review. Fourth, comparative research across different migration regimes, labor protection frameworks, and long-term care systems would enhance understanding of how policy contexts influence caregiving dynamics and outcomes. Finally, the geographic concentration of studies in this review, particularly from Asia, and specifically from Israel, highlights the need for research in underrepresented regions to improve the generalizability of findings and understand how cultural and policy contexts shape these relationships differently across diverse settings.

## Supplementary Material

gnaf300_Supplementary_Data

## Data Availability

All data, analytic methods, and study materials used in this research are available to other researchers for replication purposes. These materials can be obtained upon reasonable request from the corresponding author. Additionally, the study was pre-registered on the Open Science Framework (OSF, https://osf.io/f78j2/overview).
