# Supplementary material for: Caregiving experiences and relationships among migrant care workers, older care recipients, and employer families: a scoping review
Source: Gerontologist. 2025 Dec 15;66(3):gnaf300. doi: 10.1093/geront/gnaf300 (PMC13102631; doi:10.1093/geront/gnaf300)
Supplement: gnaf300_Supplementary_Data [file gnaf300_supplementary_data.pdf]

Caregiving Experiences and Relationships among Migrant Care Workers, Older Care Recipients and Employer Families: A Scoping Review

Igone Etxeberria PhD, Fátima María García-Pena MS, Maite Azabal MS, and Karl Pillemer PhD

Supplementary Material 1

Search Terms Used

1	**Web of Science: Core collection and Web of Science: Medline**<br>“Migrant Home Care*” (Topic) OR “Foreign Home Care*” (Topic) OR “Migrant Homecare*” (Topic) OR “Foreign Homecare*” (Topic) OR “Migrant Home Care Worker*” (Topic) OR “Foreign Home Care Worker*” (Topic) OR “Migrant Home Worker*” (Topic) OR “Foreign Home Worker*” (Topic) OR “Migrant Homeworker*” (Topic) OR “Foreign Homeworker*” (Topic) OR “Migrant Domestic Care*” (Topic) OR “Foreign Domestic Care*” (Topic) OR “Migrant Domestic Care Worker*” (Topic) OR “Foreign Domestic Care Worker*” (Topic) OR “Migrant Domestic Worker*” (Topic) OR “Foreign Domestic Worker*” (Topic) OR “In-Home Migrant Care*” (Topic) OR “In-Home Foreign Care*” (Topic) OR “In-Home Migrant Care Worker*” (Topic) OR “In-Home Foreign Care Worker*” (Topic) OR “In-Home Migrant Worker*” (Topic) OR “In-Home Foreign Worker*” (Topic) OR “In-Home Paid Migrant Care*” (Topic) OR “In-Home Foreign Paid Care*” (Topic) OR “In-Home Paid Migrant Care Worker*” (Topic) OR “In-Home Paid Foreign Care Worker*” (Topic)<br>**EBSCO Host: APA PsycInfo, EBSCO Host: Psychology and Behavioral Sciences Collection, and EBSCO Host: PsycArticles**<br>TX (“Migrant Home Care*”) OR TX (“Foreign Home Care*”) OR TX (“Migrant Homecare*”) OR TX (“Foreign Homecare*”) OR TX (“Migrant Home Care Worker*”) OR TX (“Foreign Home Care Worker*”) OR TX (“Migrant Home Worker*”) OR TX (“Foreign Home Worker*”) OR TX (“Migrant Homeworker*”) OR TX (“Foreign Homeworker*”) OR TX (“Migrant Domestic Care*”) OR TX (“Foreign Domestic Care*”) OR TX (“Migrant Domestic Care Worker*”) OR TX (“Foreign Domestic Care Worker*”) OR TX (“Migrant Domestic Worker*”) OR TX (“Foreign Domestic Worker*”) OR TX (“In-Home Migrant Care*”) OR TX (“In-Home Foreign Care*”) OR TX (“In-Home Migrant Care Worker*”) OR TX (“In-Home Foreign Care Worker*”) OR TX (“In-Home Migrant Worker*”) OR TX (“In-Home Foreign Worker*”) OR TX (“In-Home Paid Migrant Care*”) OR TX (“In-Home Foreign Paid Care*”) OR TX (“In-Home Paid Migrant Care Worker*”) OR TX (“In-Home Paid Foreign Care Worker*”)<br>**Scopus**<br>(TITLE-ABS-KEY ("Foreign Domestic Worker*")) OR (TITLE-ABS-KEY ("Migrant Domestic Worker*")) OR (TITLE-ABS-KEY ("In-Home Paid Foreign Care Worker*")) OR (TITLE-ABS-KEY ("In-Home Paid Migrant Care Worker*")) OR (TITLE-ABS-KEY ("In-Home Paid Migrant Care*")) OR (TITLE-ABS-KEY ("In-Home Foreign Worker*")) OR (TITLE-ABS-KEY ("In-Home Migrant Worker*")) OR (TITLE-ABS-KEY ("In-Home Foreign Care Worker*")) OR (TITLE-ABS-KEY ("In-Home Migrant Care Worker*")) OR (TITLE-ABS-KEY ("In-Home Foreign Care*")) OR (TITLE-ABS-KEY ("In-Home Migrant Care*")) OR (TITLE-ABS-KEY ("Foreign Domestic Care Worker*")) OR (TITLE-ABS-KEY ("Migrant Domestic Care Worker*")) OR (TITLE-ABS-KEY ("Foreign Domestic Care*")) OR (TITLE-ABS-KEY ("Migrant Domestic Care*")) OR (TITLE-ABS-KEY ("Foreign Homeworker*")) OR (TITLE-ABS-KEY ("Migrant Homeworker*")) OR (TITLE-ABS-KEY ("Foreign Home Worker*")) OR (TITLE-ABS-KEY ("Migrant

	Home Worker*")) OR (TITLE-ABS-KEY ("Foreign Home Care Worker*")) OR (TITLE-ABS-KEY ("Migrant Home Care Worker*")) OR (TITLE-ABS-KEY ("Foreign Homecare*")) OR (TITLE-ABS-KEY ("Migrant Homecare*")) OR (TITLE-ABS-KEY ("Foreign Home Care*")) OR (TITLE-ABS-KEY ("Migrant Home Care*"))<br>**PubMed**<br>((((((((((((((((((((((((("Migrant Home Care*"[Title/Abstract]) OR ("Foreign Home Care*"[Title/Abstract])) OR ("Migrant Homecare*"[Title/Abstract])) OR ("Foreign Homecare*"[Title/Abstract])) OR ("Migrant Home Care Worker*"[Title/Abstract])) OR ("Foreign Home Care Worker*"[Title/Abstract])) OR ("Migrant Home Worker*"[Title/Abstract])) OR ("Foreign Home Worker*"[Title/Abstract])) OR ("Migrant Homeworker*"[Title/Abstract])) OR ("Foreign Homeworker*"[Title/Abstract])) OR ("Migrant Domestic Care*"[Title/Abstract])) OR ("Foreign Domestic Care*"[Title/Abstract])) OR ("Migrant Domestic Care Worker*"[Title/Abstract])) OR ("Foreign Domestic Care Worker*"[Title/Abstract])) OR ("Migrant Domestic Worker*"[Title/Abstract])) OR ("Foreign Domestic Worker*"[Title/Abstract])) OR ("In-Home Migrant Care*"[Title/Abstract])) OR ("In-Home Foreign Care*"[Title/Abstract])) OR ("In-Home Migrant Care Worker*"[Title/Abstract])) OR ("In-Home Foreign Care Worker*"[Title/Abstract])) OR ("In-Home Migrant Worker*"[Title/Abstract])) OR ("In-Home Foreign Worker*"[Title/Abstract])) OR ("In-Home Paid Migrant Care*"[Title/Abstract])) OR ("In-Home Foreign Paid Care*"[Title/Abstract])) OR ("In-Home Paid Migrant Care Worker*"[Title/Abstract])) OR ("In-Home Paid Foreign Care Worker*"[Title/Abstract])<br>**Google Scholar**<br>“Migrant Home Care”; “Foreign Home Care”; “Migrant Homecare”; “Foreign Homecare”; “Migrant Home Care Worker”; “Foreign Home Care Worker”; “Migrant Home Worker”; “Foreign Home Worker”; “Migrant Homeworker”; “Foreign Homeworker”; “Migrant Domestic Care”; “Foreign Domestic Care”; “Migrant Domestic Care Worker”; “Foreign Domestic Care Worker”; “Migrant Domestic Worker”; “Foreign Domestic Worker”; “In-Home Migrant Care”; “In-Home Foreign Care”; “In-Home Migrant Care Worker”; “In-Home Foreign Care Worker”; “In-Home Migrant Worker”; “In-Home Foreign Worker”; “In-Home Paid Migrant Care”; “In-Home Foreign Paid Care”; “In-Home Paid Migrant Care Worker”; “In-Home Paid Foreign Care Worker”<br>Keywords that didn't yield results were eliminated, and a search was conducted for those that did yield results. Therefore, the search was conducted as follows:<br>(“Migrant Home Care” OR “Foreign Home Care” OR “Migrant Home Care Worker” OR “Foreign Home Care Worker” OR “Migrant Domestic Worker” OR “Foreign Domestic Worker”)
2	**Web of Science: Core collection and Web of Science: Medline**<br>“Family Care*” (Topic) OR “Employer Famil*” (Topic) OR “Informal Care*” (Topic)<br>**EBSCO Host: APA PsycInfo, EBSCO Host: Psychology and Behavioral Sciences Collection, and EBSCO Host: PsycArticles**<br>TX (“Family Care*”) OR TX (“Employer Famil*”) OR TX (“Informal Care*”)<br>**Scopus**

	TITLE-ABS-KEY ("Family Care*"); TITLE-ABS-KEY ("Employer famil*"); TITLE-ABS-KEY ("Informal Care*")<br>**PubMed**<br>(("Family Care*"[Title/Abstract]) OR ("Employer Famil*"[Title/Abstract])) OR ("Informal Care*"[Title/Abstract])<br>**Google Scholar**<br>“Family Care”; “Employer Famil”; “Informal Care”<br>Keywords that didn't yield results were eliminated, and a search was conducted for those that did yield results. Therefore, the search was conducted as follows:<br>(“Family Care” OR “Informal Care”)
3	**Web of Science: Core collection and Web of Science: Medline**<br>“Elderly” (Topic) OR “Older Adult*” (Topic) OR “Care Recipient” (Topic) OR “Dementia*” (Topic) OR “Alzheimer*” (Topic) OR “Dependen*” (Topic)<br>**EBSCO Host: APA PsycInfo, EBSCO Host: Psychology and Behavioral Sciences Collection, and EBSCO Host: PsycArticles**<br>TX (“Elderly”) OR TX (“Older Adult*”) OR TX (“Care Recipient”) OR TX (“Dementia*”) OR TX (“Alzheimer*”) OR TX (“Dependen*”)<br>**Scopus**<br>TITLE-ABS-KEY ("Elderly"); TITLE-ABS-KEY ("Older Adult*"); TITLE-ABS-KEY ("Care Recipient"); TITLE-ABS-KEY ("Dementia*"); TITLE-ABS-KEY ("Alzheimer*"); TITLE-ABS-KEY ("Dependen*")<br>**PubMed**<br>((((("Elderly"[Title/Abstract]) OR ("Older Adult*"[Title/Abstract])) OR ("Care Recipient"[Title/Abstract])) OR ("Dementia*"[Title/Abstract])) OR ("Alzheimer*"[Title/Abstract])) OR ("Dependen*"[Title/Abstract])<br>**Google Scholar**<br>“Elderly”; “Older Adult”; “Care Recipient”; “Dementia”; “Alzheimer”; “Dependen”<br>All keywords produced results, so the search terms used were:<br>(“Elderly” OR “Older Adult” OR “Care Recipient” OR “Dementia” OR “Alzheimer” OR “Dependen”)
4	**Web of Science: Core collection and Web of Science: Medline**<br># 1 AND #2<br>**EBSCO Host: APA PsycInfo, EBSCO Host: Psychology and Behavioral Sciences Collection, and EBSCO Host: PsycArticles**<br>S1 AND S2<br>**Scopus**<br>((TITLE-ABS-KEY ("Foreign Domestic Worker*")) OR (TITLE-ABS-KEY ("Migrant Domestic Worker*")) OR (TITLE-ABS-KEY ("In-Home Paid Foreign Care Worker*")) OR (TITLE-ABS-KEY ("In-Home Paid Migrant Care Worker*")) OR (TITLE-ABS-KEY ("In-Home Paid Migrant Care*")) OR (TITLE-ABS-KEY ("In-Home Foreign Worker*")) OR (TITLE-ABS-KEY ("In-Home Migrant

	Worker*")) OR (TITLE-ABS-KEY ("In-Home Foreign Care Worker*")) OR (TITLE-ABS-KEY ("In-Home Migrant Care Worker*")) OR (TITLE-ABS-KEY ("In-Home Foreign Care*")) OR (TITLE-ABS-KEY ("In-Home Migrant Care*")) OR (TITLE-ABS-KEY ("Foreign Domestic Care Worker*")) OR (TITLE-ABS-KEY ("Migrant Domestic Care Worker*")) OR (TITLE-ABS-KEY ("Foreign Domestic Care*")) OR (TITLE-ABS-KEY ("Migrant Domestic Care*")) OR (TITLE-ABS-KEY ("Foreign Homeworker*")) OR (TITLE-ABS-KEY ("Migrant Homeworker*")) OR (TITLE-ABS-KEY ("Foreign Home Worker*")) OR (TITLE-ABS-KEY ("Migrant Home Worker*")) OR (TITLE-ABS-KEY ("Foreign Home Care Worker*")) OR (TITLE-ABS-KEY ("Migrant Home Care Worker*")) OR (TITLE-ABS-KEY ("Foreign Homecare*")) OR (TITLE-ABS-KEY ("Migrant Homecare*")) OR (TITLE-ABS-KEY ("Foreign Home Care*")) OR (TITLE-ABS-KEY ("Migrant Home Care*"))) AND ((TITLE-ABS-KEY ("Informal Care*")) OR (TITLE-ABS-KEY ("Employer Famil*")) OR (TITLE-ABS-KEY ("Family Care*")))<br>**PubMed**<br>(((((((((((((((((((((((((("Migrant Home Care*"[Title/Abstract]) OR ("Foreign Home Care*"[Title/Abstract])) OR ("Migrant Homecare*"[Title/Abstract])) OR ("Foreign Homecare*"[Title/Abstract])) OR ("Migrant Home Care Worker*"[Title/Abstract])) OR ("Foreign Home Care Worker*"[Title/Abstract])) OR ("Migrant Home Worker*"[Title/Abstract])) OR ("Foreign Home Worker*"[Title/Abstract])) OR ("Migrant Homeworker*"[Title/Abstract])) OR ("Foreign Homeworker*"[Title/Abstract])) OR ("Migrant Domestic Care*"[Title/Abstract])) OR ("Foreign Domestic Care*"[Title/Abstract])) OR ("Migrant Domestic Care Worker*"[Title/Abstract])) OR ("Foreign Domestic Care Worker*"[Title/Abstract])) OR ("Migrant Domestic Worker*"[Title/Abstract])) OR ("Foreign Domestic Worker*"[Title/Abstract])) OR ("In-Home Migrant Care*"[Title/Abstract])) OR ("In-Home Foreign Care*"[Title/Abstract])) OR ("In-Home Migrant Care Worker*"[Title/Abstract])) OR ("In-Home Foreign Care Worker*"[Title/Abstract])) OR ("In-Home Migrant Worker*"[Title/Abstract])) OR ("In-Home Foreign Worker*"[Title/Abstract])) OR ("In-Home Paid Migrant Care*"[Title/Abstract])) OR ("In-Home Foreign Paid Care*"[Title/Abstract])) OR ("In-Home Paid Migrant Care Worker*"[Title/Abstract])) OR ("In-Home Paid Foreign Care Worker*"[Title/Abstract])) AND ((("Family Care*"[Title/Abstract]) OR ("Employer Famil*"[Title/Abstract])) OR ("Informal Care*"[Title/Abstract]))<br>**Google Scholar**<br>(“Migrant Home Care” OR “Foreign Home Care” OR “Migrant Home Care Worker” OR “Foreign Home Care Worker” OR “Migrant Domestic Worker” OR “Foreign Domestic Worker”) + (“Family Care” OR “Informal Care”)
5	**Web of Science: Core collection and Web of Science: Medline**<br>#1 AND #3<br>**EBSCO Host: APA PsycInfo, EBSCO Host: Psychology and Behavioral Sciences Collection, and EBSCO Host: PsycArticles**<br>S1 AND S3<br>**Scopus**<br>((TITLE-ABS-KEY ("Foreign Domestic Worker*")) OR (TITLE-ABS-KEY ("Migrant Domestic Worker*")) OR (TITLE-ABS-KEY ("In-Home Paid Foreign Care Worker*")) OR (TITLE-ABS-KEY ("In-Home Paid Migrant Care Worker*")) OR (TITLE-ABS-KEY ("In-Home Paid Migrant Care*")) OR (TITLE-ABS-KEY ("In-Home Foreign Worker*")) OR (TITLE-ABS-KEY ("In-Home Migrant

	Worker*")) OR (TITLE-ABS-KEY ("In-Home Foreign Care Worker*")) OR (TITLE-ABS-KEY ("In-Home Migrant Care Worker*")) OR (TITLE-ABS-KEY ("In-Home Foreign Care*")) OR (TITLE-ABS-KEY ("In-Home Migrant Care*")) OR (TITLE-ABS-KEY ("Foreign Domestic Care Worker*")) OR (TITLE-ABS-KEY ("Migrant Domestic Care Worker*")) OR (TITLE-ABS-KEY ("Foreign Domestic Care*")) OR (TITLE-ABS-KEY ("Migrant Domestic Care*")) OR (TITLE-ABS-KEY ("Foreign Homeworker*")) OR (TITLE-ABS-KEY ("Migrant Homeworker*")) OR (TITLE-ABS-KEY ("Foreign Home Worker*")) OR (TITLE-ABS-KEY ("Migrant Home Worker*")) OR (TITLE-ABS-KEY ("Foreign Home Care Worker*")) OR (TITLE-ABS-KEY ("Migrant Home Care Worker*")) OR (TITLE-ABS-KEY ("Foreign Homecare*")) OR (TITLE-ABS-KEY ("Migrant Homecare*")) OR (TITLE-ABS-KEY ("Foreign Home Care*")) OR (TITLE-ABS-KEY ("Migrant Home Care*"))) AND ((TITLE-ABS-KEY ("Dependen*")) OR (TITLE-ABS-KEY ("Alzheimer*")) OR (TITLE-ABS-KEY ("Dementia*")) OR (TITLE-ABS-KEY ("Care Recipient")) OR (TITLE-ABS-KEY ("Older Adult*")) OR (TITLE-ABS-KEY ("Elderly"))))<br>**PubMed**<br>((((((((((((((((((((((("Migrant Home Care*"[Title/Abstract]) OR ("Foreign Home Care*"[Title/Abstract])) OR ("Migrant Homecare*"[Title/Abstract])) OR ("Foreign Homecare*"[Title/Abstract])) OR ("Migrant Home Care Worker*"[Title/Abstract])) OR ("Foreign Home Care Worker*"[Title/Abstract])) OR ("Migrant Home Worker*"[Title/Abstract])) OR ("Foreign Home Worker*"[Title/Abstract])) OR ("Migrant Homeworker*"[Title/Abstract])) OR ("Foreign Homeworker*"[Title/Abstract])) OR ("Migrant Domestic Care*"[Title/Abstract])) OR ("Foreign Domestic Care*"[Title/Abstract])) OR ("Migrant Domestic Care Worker*"[Title/Abstract])) OR ("Foreign Domestic Care Worker*"[Title/Abstract])) OR ("Migrant Domestic Worker*"[Title/Abstract])) OR ("Foreign Domestic Worker*"[Title/Abstract])) OR ("In-Home Migrant Care*"[Title/Abstract])) OR ("In-Home Foreign Care*"[Title/Abstract])) OR ("In-Home Migrant Care Worker*"[Title/Abstract])) OR ("In-Home Foreign Care Worker*"[Title/Abstract])) OR ("In-Home Migrant Worker*"[Title/Abstract])) OR ("In-Home Foreign Worker*"[Title/Abstract])) OR ("In-Home Paid Migrant Care*"[Title/Abstract])) OR ("In-Home Foreign Paid Care*"[Title/Abstract])) OR ("In-Home Paid Migrant Care Worker*"[Title/Abstract])) OR ("In-Home Paid Foreign Care Worker*"[Title/Abstract])) AND (((((("Elderly"[Title/Abstract]) OR ("Older Adult*"[Title/Abstract])) OR ("Care Recipient"[Title/Abstract])) OR ("Dementia*"[Title/Abstract])) OR ("Alzheimer*"[Title/Abstract])) OR ("Dependen*"[Title/Abstract]))<br>**Google Scholar**<br>(“Migrant Home Care” OR “Foreign Home Care” OR “Migrant Home Care Worker” OR “Foreign Home Care Worker” OR “Migrant Domestic Worker” OR “Foreign Domestic Worker”) + (“Elderly” OR “Older Adult” OR “Care Recipient” OR “Dementia” OR “Alzheimer” OR “Dependen”)
6	**Web of Science: Core collection**<br>#4 OR #5<br>Filters applied: Publication years: 2013-2025; Document types: exclude review article, book chapters, and editorial material.<br>**Web of Science: Medline**<br>#4 OR #5<br>Filters applied: Publication years: 2013-2025; Document types: exclude editorial, letter, and systematic review

	**EBSCO Host: APA PsycInfo**<br>S4 OR S5<br>Filters applied: Publication date: 01/01/2013-12/09/2025; Source types: academic journals; Language: English, Spanish; Castilian<br>**EBSCO Host: Psychology and Behavioral Sciences Collection**<br>S4 OR S5<br>Filters applied: Publication date: 01/01/2013-12/09/2025; Source types: academic journal; Language: English<br>**EBSCO Host: PsycArticles**<br>S4 OR S5<br>Filters applied: Publication date: 01/01/2013-12/09/2025; Source types: all journals, peer reviewed journals; Language: English<br>**Scopus**<br>(((TITLE-ABS-KEY ("Foreign Domestic Worker*")) OR (TITLE-ABS-KEY ("Migrant Domestic Worker*")) OR (TITLE-ABS-KEY ("In-Home Paid Foreign Care Worker*")) OR (TITLE-ABS-KEY ("In-Home Paid Migrant Care Worker*")) OR (TITLE-ABS-KEY ("In-Home Paid Migrant Care*")) OR (TITLE-ABS-KEY ("In-Home Foreign Worker*")) OR (TITLE-ABS-KEY ("In-Home Migrant Worker*")) OR (TITLE-ABS-KEY ("In-Home Foreign Care Worker*")) OR (TITLE-ABS-KEY ("In-Home Migrant Care Worker*")) OR (TITLE-ABS-KEY ("In-Home Foreign Care*")) OR (TITLE-ABS-KEY ("In-Home Migrant Care*")) OR (TITLE-ABS-KEY ("Foreign Domestic Care Worker*")) OR (TITLE-ABS-KEY ("Migrant Domestic Care Worker*")) OR (TITLE-ABS-KEY ("Foreign Domestic Care*")) OR (TITLE-ABS-KEY ("Migrant Domestic Care*")) OR (TITLE-ABS-KEY ("Foreign Homeworker*")) OR (TITLE-ABS-KEY ("Migrant Homeworker*")) OR (TITLE-ABS-KEY ("Foreign Home Worker*")) OR (TITLE-ABS-KEY ("Migrant Home Worker*")) OR (TITLE-ABS-KEY ("Foreign Home Care Worker*")) OR (TITLE-ABS-KEY ("Migrant Home Care Worker*")) OR (TITLE-ABS-KEY ("Foreign Homecare*")) OR (TITLE-ABS-KEY ("Migrant Homecare*")) OR (TITLE-ABS-KEY ("Foreign Home Care*")) OR (TITLE-ABS-KEY ("Migrant Home Care*"))) AND ((TITLE-ABS-KEY ("Dependen*")) OR (TITLE-ABS-KEY ("Alzheimer*")) OR (TITLE-ABS-KEY ("Dementia*")) OR (TITLE-ABS-KEY ("Care Recipient")) OR (TITLE-ABS-KEY ("Older Adult*")) OR (TITLE-ABS-KEY ("Elderly")))) OR (((TITLE-ABS-KEY ("Foreign Domestic Worker*")) OR (TITLE-ABS-KEY ("Migrant Domestic Worker*")) OR (TITLE-ABS-KEY ("In-Home Paid Foreign Care Worker*")) OR (TITLE-ABS-KEY ("In-Home Paid Migrant Care Worker*")) OR (TITLE-ABS-KEY ("In-Home Paid Migrant Care*")) OR (TITLE-ABS-KEY ("In-Home Foreign Worker*")) OR (TITLE-ABS-KEY ("In-Home Migrant Worker*")) OR (TITLE-ABS-KEY ("In-Home Foreign Care Worker*")) OR (TITLE-ABS-KEY ("In-Home Migrant Care Worker*")) OR (TITLE-ABS-KEY ("In-Home Foreign Care*")) OR (TITLE-ABS-KEY ("In-Home Migrant Care*")) OR (TITLE-ABS-KEY ("Foreign Domestic Care Worker*")) OR (TITLE-ABS-KEY ("Migrant Domestic Care Worker*")) OR (TITLE-ABS-KEY ("Foreign Domestic Care*")) OR (TITLE-ABS-KEY ("Migrant Domestic Care*")) OR (TITLE-ABS-KEY ("Foreign Homeworker*")) OR (TITLE-ABS-KEY ("Migrant Homeworker*")) OR (TITLE-ABS-KEY ("Foreign Home Worker*")) OR (TITLE-ABS-KEY ("Migrant Home Worker*")) OR (TITLE-ABS-KEY ("Foreign Home Care Worker*")) OR (TITLE-ABS-KEY ("Migrant Home Care Worker*")) OR (TITLE-ABS-KEY ("Foreign Homecare*")) OR (TITLE-ABS-KEY ("Migrant Homecare*")) OR (TITLE-ABS-KEY ("Foreign Home Care*")) OR (TITLE-ABS-KEY ("Migrant Home Care*"

	))) AND ((TITLE-ABS-KEY ("Informal Care*")) OR (TITLE-ABS-KEY ("Employer Famil*")) OR (TITLE-ABS-KEY ("Family Care*"))))<br>Filters applied: Year: 2013-2025; Document type: article; Language: English<br>**PubMed**<br>((((((((((((((((((((((((((("Migrant Home Care*"[Title/Abstract]) OR ("Foreign Home Care*"[Title/Abstract])) OR ("Migrant Homecare*"[Title/Abstract])) OR ("Foreign Homecare*"[Title/Abstract])) OR ("Migrant Home Care Worker*"[Title/Abstract])) OR ("Foreign Home Care Worker*"[Title/Abstract])) OR ("Migrant Home Worker*"[Title/Abstract])) OR ("Foreign Home Worker*"[Title/Abstract])) OR ("Migrant Homeworker*"[Title/Abstract])) OR ("Foreign Homeworker*"[Title/Abstract])) OR ("Migrant Domestic Care*"[Title/Abstract])) OR ("Foreign Domestic Care*"[Title/Abstract])) OR ("Migrant Domestic Care Worker*"[Title/Abstract])) OR ("Foreign Domestic Care Worker*"[Title/Abstract])) OR ("Migrant Domestic Worker*"[Title/Abstract])) OR ("Foreign Domestic Worker*"[Title/Abstract])) OR ("In-Home Migrant Care*"[Title/Abstract])) OR ("In-Home Foreign Care*"[Title/Abstract])) OR ("In-Home Migrant Care Worker*"[Title/Abstract])) OR ("In-Home Foreign Care Worker*"[Title/Abstract])) OR ("In-Home Migrant Worker*"[Title/Abstract])) OR ("In-Home Foreign Worker*"[Title/Abstract])) OR ("In-Home Paid Migrant Care*"[Title/Abstract])) OR ("In-Home Foreign Paid Care*"[Title/Abstract])) OR ("In-Home Paid Migrant Care Worker*"[Title/Abstract])) OR ("In-Home Paid Foreign Care Worker*"[Title/Abstract])) AND ((("Family Care*"[Title/Abstract]) OR ("Employer Famil*"[Title/Abstract])) OR ("Informal Care*"[Title/Abstract]))) OR ((((((((((((((((((((((((((("Migrant Home Care*"[Title/Abstract]) OR ("Foreign Home Care*"[Title/Abstract])) OR ("Migrant Homecare*"[Title/Abstract])) OR ("Foreign Homecare*"[Title/Abstract])) OR ("Migrant Home Care Worker*"[Title/Abstract])) OR ("Foreign Home Care Worker*"[Title/Abstract])) OR ("Migrant Home Worker*"[Title/Abstract])) OR ("Foreign Home Worker*"[Title/Abstract])) OR ("Migrant Homeworker*"[Title/Abstract])) OR ("Foreign Homeworker*"[Title/Abstract])) OR ("Migrant Domestic Care*"[Title/Abstract])) OR ("Foreign Domestic Care*"[Title/Abstract])) OR ("Migrant Domestic Care Worker*"[Title/Abstract])) OR ("Foreign Domestic Care Worker*"[Title/Abstract])) OR ("Migrant Domestic Worker*"[Title/Abstract])) OR ("Foreign Domestic Worker*"[Title/Abstract])) OR ("In-Home Migrant Care*"[Title/Abstract])) OR ("In-Home Foreign Care*"[Title/Abstract])) OR ("In-Home Migrant Care Worker*"[Title/Abstract])) OR ("In-Home Foreign Care Worker*"[Title/Abstract])) OR ("In-Home Migrant Worker*"[Title/Abstract])) OR ("In-Home Foreign Worker*"[Title/Abstract])) OR ("In-Home Paid Migrant Care*"[Title/Abstract])) OR ("In-Home Foreign Paid Care*"[Title/Abstract])) OR ("In-Home Paid Migrant Care Worker*"[Title/Abstract])) OR ("In-Home Paid Foreign Care Worker*"[Title/Abstract])) AND (((((("Elderly"[Title/Abstract]) OR ("Older Adult*"[Title/Abstract])) OR ("Care Recipient"[Title/Abstract])) OR ("Dementia*"[Title/Abstract])) OR ("Alzheimer*"[Title/Abstract])) OR ("Dependen*"[Title/Abstract])))<br>Filters applied: Publication date: 01/01/2013-12/09/2025<br>**Google Scholar**<br>(“Migrant Home Care” OR “Foreign Home Care” OR “Migrant Home Care Worker” OR “Foreign Home Care Worker” OR “Migrant Domestic Worker” OR “Foreign Domestic Worker”) + (“Family Care” OR “Informal Care”) + (“Migrant Home Care” OR “Foreign Home Care” OR “Migrant Home Care Worker” OR “Foreign Home Care Worker” OR “Migrant Domestic Worker” OR “Foreign Domestic Worker”) + (“Elderly” OR “Older Adult” OR “Care Recipient” OR “Dementia” OR “Alzheimer” OR “Dependen”)

	Filters applied: Data: 2013-2025; Any type; Not include citations

Supplementary Material 2

Transparency Acknowledgement Appendix

Country	Author(s)	Number of studies	Notes
Israel	Ayalon and colleagues	8	There is a high concentration of studies from the same country (*n* = 16), and most were conducted by the same research groups (*n* = 8).
	Cohen-Mansfield & Golander	4	
	Iecovich	1	
	Goldweig et al.	1	
	Mazuz	1	
	Zriker et al.	1	
Singapore	Yeoh, Liew and colleagues	2	There is a high concentration of studies from the same country (*n* = 9).
	Basnyat and Chang	1	
	Heng et al.	1	
	Kaur-Gill and Dutta	1	
	Mehta and Leng	1	
	Østbye et al.	1	
	Tam et al.	1	
	Yuan et al.	1	
Hong Kong	Ho et al.*	4	There is a high concentration of studies from the same country (*n* = 6), and most were conducted by the same research groups (*n* = 4).
	Lai and Fong	1	
	Hung et al.	1	
Taiwan	Munkejord et al.	2	The distribution of the articles across countries is more varied.
	Septi-Mauludina et al.	1	
Germany	Kriegsman-Rabe et al.	1	
	Grenz and von Kutzleben	1	
Belgium	Hoens and Semtcoren	1	
Netherlands	van Bochove and zur Kleinsmiede	1	

Note. *One of these studies also includes the United Kingdom.

Supplementary Material 3

Preferred Reporting Items for Systematic reviews and Meta-Analyses extension for Scoping Reviews (PRISMA-ScR) Checklist

SECTION	ITEM	PRISMA-ScR CHECKLIST ITEM	REPORTED ON PAGE #
**TITLE**			
Title	1	Identify the report as a scoping review.	1
**ABSTRACT**			
Structured summary	2	Provide a structured summary that includes (as applicable): background, objectives, eligibility criteria, sources of evidence, charting methods, results, and conclusions that relate to the review questions and objectives.	3-4
**INTRODUCTION**			
Rationale	3	Describe the rationale for the review in the context of what is already known. Explain why the review questions/objectives lend themselves to a scoping review approach.	5-8
Objectives	4	Provide an explicit statement of the questions and objectives being addressed with reference to their key elements (e.g., population or participants, concepts, and context) or other relevant key elements used to conceptualize the review questions and/or objectives.	8
**METHODS**			
Protocol and registration	5	Indicate whether a review protocol exists; state if and where it can be accessed (e.g., a Web address); and if available, provide registration information, including the registration number.	9
Eligibility criteria	6	Specify characteristics of the sources of evidence used as eligibility criteria (e.g., years considered, language, and publication status), and provide a rationale.	9
Information sources*	7	Describe all information sources in the search (e.g., databases with dates of coverage and contact with authors to identify additional sources), as well as the date the most recent search was executed.	9-10
Search	8	Present the full electronic search strategy for at least 1 database, including any limits used, such that it could be repeated.	Table 1 and Supplementary material 1

SECTION	ITEM	PRISMA-ScR CHECKLIST ITEM	REPORTED ON PAGE #
Selection of sources of evidence†	9	State the process for selecting sources of evidence (i.e., screening and eligibility) included in the scoping review.	9-10
Data charting process‡	10	Describe the methods of charting data from the included sources of evidence (e.g., calibrated forms or forms that have been tested by the team before their use, and whether data charting was done independently or in duplicate) and any processes for obtaining and confirming data from investigators.	10-11
Data items	11	List and define all variables for which data were sought and any assumptions and simplifications made.	11-12
Critical appraisal of individual sources of evidence§	12	If done, provide a rationale for conducting a critical appraisal of included sources of evidence; describe the methods used and how this information was used in any data synthesis (if appropriate).	N/A
Synthesis of results	13	Describe the methods of handling and summarizing the data that were charted.	11-12
**RESULTS**			
Selection of sources of evidence	14	Give numbers of sources of evidence screened, assessed for eligibility, and included in the review, with reasons for exclusions at each stage, ideally using a flow diagram.	12, Figure 1
Characteristics of sources of evidence	15	For each source of evidence, present characteristics for which data were charted and provide the citations.	12-14
Critical appraisal within sources of evidence	16	If done, present data on critical appraisal of included sources of evidence (see item 12).	N/A
Results of individual sources of evidence	17	For each included source of evidence, present the relevant data that were charted that relate to the review questions and objectives.	Table 2
Synthesis of results	18	Summarize and/or present the charting results as they relate to the review questions and objectives.	14-25
**DISCUSSION**			
Summary of evidence	19	Summarize the main results (including an overview of concepts, themes, and types of evidence available), link to the review	25-30

SECTION	ITEM	PRISMA-ScR CHECKLIST ITEM	REPORTED ON PAGE #
		questions and objectives, and consider the relevance to key groups.	
Limitations	20	Discuss the limitations of the scoping review process.	30-31
Conclusions	21	Provide a general interpretation of the results with respect to the review questions and objectives, as well as potential implications and/or next steps.	31-34
**FUNDING**			
Funding	22	Describe sources of funding for the included sources of evidence, as well as sources of funding for the scoping review. Describe the role of the funders of the scoping review.	Title page

JBI = Joanna Briggs Institute; PRISMA-ScR = Preferred Reporting Items for Systematic reviews and Meta-Analyses extension for Scoping Reviews.
* Where *sources of evidence* (see second footnote) are compiled from, such as bibliographic databases, social media platforms, and Web sites.
† A more inclusive/heterogeneous term used to account for the different types of evidence or data sources (e.g., quantitative and/or qualitative research, expert opinion, and policy documents) that may be eligible in a scoping review as opposed to only studies. This is not to be confused with *information sources* (see first footnote).
‡ The frameworks by Arksey and O'Malley (6) and Levac and colleagues (7) and the JBI guidance (4, 5) refer to the process of data extraction in a scoping review as data charting.
§ The process of systematically examining research evidence to assess its validity, results, and relevance before using it to inform a decision. This term is used for items 12 and 19 instead of "risk of bias" (which is more applicable to systematic reviews of interventions) to include and acknowledge the various sources of evidence that may be used in a scoping review (e.g., quantitative and/or qualitative research, expert opinion, and policy document).

From: Tricco AC, Lillie E, Zarin W, O'Brien KK, Colquhoun H, Levac D, et al. PRISMA Extension for Scoping Reviews (PRISMAScR): Checklist and Explanation. Ann Intern Med. 2018;169:467–473. doi: 10.7326/M18-0850.
